# Population Genomics of the Immune Evasion *(var)* Genes of Plasmodium falciparum

**DOI:** 10.1371/journal.ppat.0030034

**Published:** 2007-03-16

**Authors:** Alyssa E Barry, Aleksandra Leliwa-Sytek, Livingston Tavul, Heather Imrie, Florence Migot-Nabias, Stuart M Brown, Gilean A. V McVean, Karen P Day

**Affiliations:** 1 Peter Medawar Building for Pathogen Research, University of Oxford, Oxford, United Kingdom; 2 Department of Zoology, University of Oxford, Oxford, United Kingdom; 3 Papua New Guinea Institute for Medical Research, Madang, Papua New Guinea; 4 Centre International de Recherches Médicales de Franceville (CIRMF), Franceville, Gabon; 5 Research Computing Resource, New York University School of Medicine, New York, New York, United States of America; 6 Department of Statistics, University of Oxford, Oxford, United Kingdom; University of Cambridge, United Kingdom

## Abstract

*Var* genes encode the major surface antigen (PfEMP1) of the blood stages of the human malaria parasite *Plasmodium falciparum.* Differential expression of up to 60 diverse *var* genes in each parasite genome underlies immune evasion. We compared the diversity of the DBLα domain of *var* genes sampled from 30 parasite isolates from a malaria endemic area of Papua New Guinea (PNG) and 59 from widespread geographic origins (global). Overall, we obtained over 8,000 quality-controlled DBLα sequences. Within our sampling frame, the global population had a total of 895 distinct DBLα “types” and negligible overlap among repertoires. This indicated that *var* gene diversity on a global scale is so immense that many genomes would need to be sequenced to capture its true extent. In contrast, we found a much lower diversity in PNG of 185 DBLα types, with an average of approximately 7% overlap among repertoires. While we identify marked geographic structuring, nearly 40% of types identified in PNG were also found in samples from different countries showing a cosmopolitan distribution for much of the diversity. We also present evidence to suggest that recombination plays a key role in maintaining the unprecedented levels of polymorphism found in these immune evasion genes. This population genomic framework provides a cost effective molecular epidemiological tool to rapidly explore the geographic diversity of *var* genes.

## Author Summary

Malaria parasites live in red blood cells of the human host for part of the life cycle, during which a family of diverse antigens known as PfEMP1 are placed on the surface. PfEMP1 variants switch by sequential expression of up to 60 *var* genes. This allows the parasite to evade immune detection within an individual host, enhancing its chances to be transmitted to the mosquito vector in situations where mosquitoes are seasonally available. Methods to rapidly assess *var* gene diversity in parasite populations are needed to measure antigenic diversity and define relationships with malaria transmission. Using a specialized framework, we completed the first systematic sampling of *var* genes from parasite genomes obtained from the same (Papua New Guinea [PNG]) and different (global) populations. Globally, there was no limit to the number of *var* genes because parasites rarely shared *var* genes. In PNG, *var* gene numbers were restricted due to high levels of sharing, and most were only found in that population. Recombination was important to the evolution of *var* genes in PNG. The data suggest there are distinct *var* genes in different populations, which may have consequences for the spread of malarial disease from one geographic area to another.

## Introduction

Quantifying the diversity of major surface antigens underlying immune evasion of HIV 1 and 2 and Influenza A has been central to characterizing the transmission dynamics of these important human pathogens. In addition, documentation of variation data has provided a basis for the development of candidate vaccine targets [1,2]. Surprisingly, in-depth molecular epidemiological sampling and population genomic analyses of the *var* genes encoding the major blood stage surface antigen of the malaria parasite, Plasmodium falciparum erythrocyte membrane protein 1 (PfEMP1), has not been done. This is largely due to the inherent difficulties in the population genomic analysis of highly diverse multigene families. We set out to develop and evaluate a rapid, molecular epidemiological population genomic framework to investigate *var* gene diversity in natural parasite populations, due to the importance of these genes to the biology of *P. falciparum.*

To achieve chronic infection, malaria parasites evade the host immune response by switching PfEMP1 isoforms through differential expression of members of the *var* multigene family [3–5]. PfEMP1 is expressed on the surface of blood stage parasites known as trophozoites [6] and the transmission (early gametocyte) stages [7]. Parasite adhesion occurs in the deep vasculature of host tissues by binding of PfEMP1 to host endothelial cell receptors. Some PfEMP1-adhesion interactions are proposed to lead to severe disease manifestations such as cerebral and placental malaria (reviewed in [8]). Variant specific anti-PfEMP1 antibodies are believed to contribute to the regulation of parasite density in a manner that decreases the incidence of clinical disease [9–13]. This immunity may reduce the duration of infection in a variant-specific manner to drive the dynamics of multiple infections in semi-immune children [14] and induced infections in humans [15]. Immunity to PfEMP1 can thereby influence transmission by reducing persistence. It may also reduce transmission by regulating the density of asexual blood stages with potential to become transmission stages and by directly targeting early gametocytes to prevent the maturation of transmission stages [16]. Consequently, diversity of PfEMP1 or *var* genes is able to promote transmission success by immune evasion.

Describing the diversity of *var* genes presents a more complex problem than assessing the diversity of the single copy major surface antigen genes of HIV and Influenza A. Individual *P. falciparum* genomes have repertoires of *var* genes that can recombine with other repertoires during the obligatory sexual phase of the life cycle in the mosquito [17–19]. There is also circumstantial evidence for ectopic recombination among *var* genes within the same genome, possibly during both meiosis and mitosis [20–22]. Therefore, there is enormous potential to generate diversity, even among closely related genomes. *Var* genes are large (5–12 kb) and complex [23–26], encoding variable numbers and classes of the adhesion domains, Duffy binding like (DBL: α, β, δ, ɛ, and γ classes) and cysteine interdomain rich (CIDR: α, β, and γ classes) [26]. Both size and complexity preclude population genomic analysis of the full *var* gene sequences. The DBLα encoding domain was used previously as a marker of *var* genes in investigations of diversity [21,22,27–30] and expression [31–33]. The small size (∼1 kb) and ubiquitous presence of DBLα among *var* genes [24–26,34] make this domain a suitable population genomic marker.

Previous analyses of *var* gene diversity have examined DBLα domain sequences from the *var* gene repertoires of allopatric (distantly related) [22,27,30] or just a few sympatric (closely related) [28,29] isolates. These studies have established that any pair of DBLα sequences from the same genome were on average as diverse as any pair from two different genomes, with a range of 45%–100% amino acid identity [3,22,27,28]. This has made it impossible to identify *var* gene orthologs among genomes. Limited overlap (shared DBLα sequences) among *var* repertoires from sympatric isolates has also been reported [27–29], suggesting that many genomes must be sampled to see the extent of diversity of these genes in natural populations. Given the importance of *var* genes to transmission, a systematic sequencing effort and population genomic analysis is needed to examine *var* gene diversity in sufficient depth to estimate levels of antigenic diversity within natural populations.

**Figure 1:**
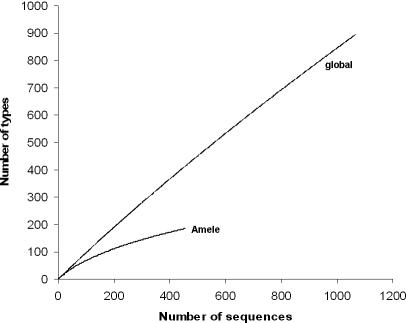
Cumulative Diversity Curves for DBLα from the Amele and Global Populations The cumulative diversity of DBLα was determined by comparing each new DBLα repertoire to previous repertoire(s) using a 96% DNA sequence identity cut-off. We plotted the number of accumulating “types” (distinct sequences) against the number of “sequences” compared. For example, the first point on the plot will be the number of types found in both isolates A and B on the y-axis against the total number of sequences compared on the x-axis. The next point will be the number of types found in isolates A, B, and C against the total number of sequences compared. The plot shown here is the average curve of 1,000 permutations of the order that isolates were compared (i.e., isolates A, B, then C; or A, C, then B; or B, C, then A).

A high-throughput population genomic framework was developed to address sampling issues specific to the molecular epidemiological analysis of diverse multigene families. This allowed the random sampling of the *var* gene repertoires of culture-adapted and field isolates of P. falciparum by sequencing DBLα domains as population genomic markers (Figure 1). *Var* genes were sampled from a “global” collection of isolates, including clones 3D7 and HB3 used in genome sequencing projects ([34]; D. Wirth, personal communication). DBLα sequences from these isolates were used to validate the framework. The “global” DBLα sequences were combined with available data from previous studies and compared to that obtained from a local population of Papua New Guinea (PNG). The results show immense levels of diversity among the *var* genes with strong evidence of geographic structuring of variation. We demonstrate patterns of similarity among sequences that suggest the widespread action of recombination in creating and maintaining diversity.

## Results

### Population Sampling

We tested a population genomic framework (Figures S1–S4; Text S1) to sample and analyze the *var* gene sequences of 25 cloned P. falciparum lines/isolates from widespread geographic origins (global; see Materials and Methods). This gave 4,754 reads (median = 161.5; range = 43–311 per isolate) of high quality DBLα sequences, which after removing redundancy, resulted in 608 consensus sequences (median = 20; range = 10–53 per isolate), with an average of seven times coverage. The GenBank accession numbers for these sequences can be found in Table S1. Artifacts were limited due to the multiple reads contributing to each DBLα sequence and by the use of a 96% DNA sequence identity cut-off (grouping reads from a single isolate with less than 4% sequence differences into a single consensus). To calculate the number of distinct DBLα sequences among isolates, individual repertoires were compared with each other, again using an arbitrary 96% DNA sequence identity cut-off. This identified shared DBLα sequences (i.e., overlap) among *var* repertoires. It also defined all of the distinct DBLα sequences in the global population sample. We named these distinct sequences “DBLα types” rather than alleles because the possibility of ectopic recombination means that the ancestry of a particular *var* gene sequence will be unlikely to be linked to physical location. It should be noted that by defining types in this way, synonymous and nonsynonymous mutations were treated equally. We identified 534 distinct DBLα types from the 608 sequences obtained from the global isolates. This large number of types confirmed the wide-ranging amplification capacity of the degenerate primers (see Materials and Methods, Text S2). Some *var* gene types were shared among isolates as shown by the fact that the number of DBLα sequences was greater than the number of DBLα types in the population. When 480 DBLα sequences (404 types) obtained from the GenBank database (see Materials and Methods) were combined with these data, 1,088 DBLα sequences and 895 DBLα types were defined. We did not have information for the number of reads contributing to this additional data.

Local sampling of 30 P. falciparum isolates from the population of Amele, PNG (see Materials and Methods) gave 3,080 reads (median = 85.5; range = 8–238 per isolate). Analysis of the sequence data resulted in 460 DBLα sequences (median = 15; range = 2–30 per isolate) with each sequence having an average of 6.7 times coverage. The GenBank accession numbers for these sequences can be found in Table S1. We defined 185 DBLα types from the sequences obtained. The ratio of sequences to types for the global sampling (ratio = 1.21) was much less than that of Amele local sampling (ratio = 2.5), demonstrating greater overlap amongst *var* gene repertoires from the Amele isolates than those from the global collection. This overlap is defined more precisely below.

### Cumulative Diversity

An important question in malaria population genomics is “how many isolates need to be sampled to find the majority of *var* gene diversity in a population?” To answer this question, we applied an ecological method to the data. We measured how the rate of DBLα type discovery changed with sample size in a manner similar to a species richness curve [35]. This method was previously used to measure heterozygosity in the human blood group antigens [36]. The number of DBLα types was plotted against the number of DBLα sequences as the *var* gene repertoire from each isolate was sampled. For sampling of global isolates, the curve did not plateau even though we sampled more than 1,000 DBLα sequences from 59 isolates (the 25 global isolates we sampled plus 34 *var* repertoires available from GenBank including the entire 3D7 *var* repertoire), showing that the majority of the *var* gene diversity in the global P. falciparum population was not defined (Figure 1). When we plotted the cumulative diversity for the Amele local population, the curve began to deviate from that of the global sample after sampling just 50 DBLα sequences (approximately three isolates) and tended toward a plateau after sampling 460 DBLα sequences (30 isolates). Thus, while *var* gene diversity was immense on a global scale it was restricted in this local population of PNG. The shape of the curve suggested that a large proportion (around 50%–60%) of common types were sampled in the local population of Amele, although this may not represent all of the diversity in the Madang region or in PNG.

### Pairwise Type Sharing

**Figure 2:**
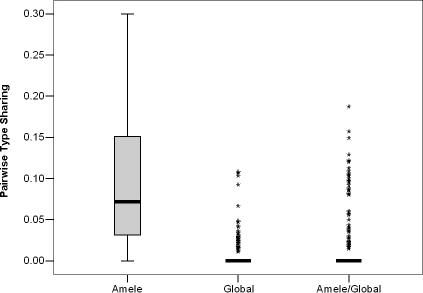
PTS among DBLα Repertoires from Amele and Global Populations PTS was plotted for pairs of Amele DBLα repertoires, global DBLα repertoires, and Amele DBLα repertoires paired with global DBLα repertoires. The median (thick line), interquartile range (boxes), approximate 95% sampling interval (whiskers = 1.5 times the interquartile range), and suspected outliers (asterisks) are shown. The raw data for these plots can be found in Figure S5.

The higher ratio of DBLα sequences to DBLα types and the flattening of the cumulative curve for Amele in comparison to that for global indicated a greater degree of *var* repertoire overlap among Amele isolates than among global isolates. To quantify this overlap in the two populations, we considered the proportion of those types that were found in both of a pair of isolates; a statistic designated the pairwise type sharing (PTS) (see Materials and Methods). We found that average PTS among Amele isolates (or within the population) was significantly greater than for global isolates (Figures 2 and S5). The PTS statistic estimated that 7% of *var* genes were shared among Amele isolates, but that a negligible number of *var* genes were shared among global isolates. When the two populations were compared, again the PTS was negligible (Figure 2). Note that we removed other PNG isolates from the global population (see Materials and Methods) for this analysis as these showed significantly higher PTS values with Amele isolates (unpublished data). The higher degree of overlap in Amele in relation to that among global isolates demonstrated the presence of geographic population structure. In particular, there was higher overlap among the contemporary Amele isolates and among those isolates and PNG isolates, also from Amele, but collected at different time periods (see Materials and Methods). Some of this will be due to the presence of a few high-frequency types in Amele (Table S2).

### Geographic Distribution of Amele Types

To test whether there was any geographic population structure, we examined the distribution of Amele types in the global population sample. The majority of the defined Amele types (61.6%) was unique to PNG. The remaining 71 types were found in Africa (24.8%), Asia (3.2%), and Latin America (3.8%); two were found in all malaria endemic regions and ten types were found in either Africa and Asia (2.2%); Africa and Latin America (2.7%); or Asia and Latin America (0.5%) (Table S2). The geographic restriction of Amele types to PNG demonstrates the existence of geographic population structure. Nevertheless, a substantial fraction of types has a cosmopolitan distribution indicating that different geographic regions do not have completely different repertoires of *var* genes.

### Sequence Diversity among Amele Types

**Figure 3:**
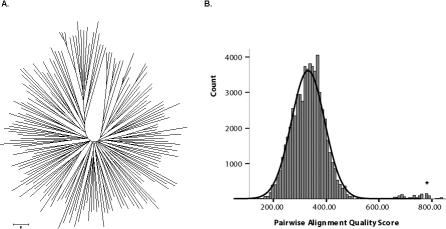
Diversity of Amele DBLα Sequences Relationships among the *var* gene sequences from Amele were defined by (A) phylogenetic analysis of Amele DBLα types. A multiple alignment of amino acid sequences was constructed in Clustal W [70] with parameters set at gap opening = 5 and gap extension = 0.05, for all translated DBLα types (*n* = 177 after removal of those with frame shifts) in the Amele population. A neighbor-joining tree was constructed in MEGA version 3.0 [71] for Amele types using the p-distance algorithm, pairwise alignment, and 1,000 bootstrapping repetitions. The consensus tree is shown. (B) Analysis of the pairwise alignment quality scores for Amele DBLα amino acid sequences. Here, the frequency distribution of scores is shown. Note the approximately Normal distribution of scores for the cluster of scores to the left, i.e., the dark line indicates maximum likelihood estimate for the Normal distribution. The asterisk indicates a cluster of pairs with high quality scores, which correspond to the shared sequences among isolates (types).

Having demonstrated extremely high levels of diversity among the *var* gene sequences, we wanted to explore potential structuring within the data. For example, do sequences broadly fall into a few groups? Or is every sequence equally different from all others? A natural approach to analyzing such structure within the data is to construct a phylogenetic tree for the sequences from a multiple-sequence alignment of the translated DNA sequences (Figure S6). The phylogeny estimated from the aligned DBLα types from Amele showed mostly distant relationships with long external branches and short, unresolved internal branches (Figure 3A). This “star-like” phylogeny suggests that the sequences are so diverse that little structuring to the variation is apparent. One evolutionary force known to result in “star-like” trees is recombination [37], suggesting that frequent exchange between different *var* gene copies may be occurring. This tree shape may also result from a rapidly expanding population or other forms of natural selection [37]. However, it is also possible that the “star-like” quality may simply reflect a low quality multiple sequence alignment (Figure S6). This showed it was difficult to assess the relatedness of *var* gene sequences using multiple sequence alignments. Alternatively, pairwise analyses will maximize the quality of an alignment and highlight the most highly related fragments of a pair. Quality scores from these alignments are better estimates of the relationship among potentially recombined sequences because they are based on the percent similarity as well as the length of the alignment (i.e., the partial alignment of a pair of recombined sequences will have a higher score than a pair of sequences with an equal number of polymorphisms across their entire length; see Materials and Methods). The distribution of the quality scores can then be used to identify potential clustering of even weakly related sequences. Therefore, we also considered the distribution of the quality scores from pairwise sequence alignments.

Pairwise alignments of the translated DBLα sequences (i.e., prior to type definition) showed an average amino acid similarity of 66% (range = 22%–100%). The distribution of quality scores among Amele DBLα sequences showed a bimodal distribution with a small cluster of high scoring alignments to the right (Figure 3B). These clusters corresponded to the sequences that were shared among isolates (i.e., types) and those that had a high degree of similarity. The majority of sequences, however, show more distant relationships, resulting in a distribution of quality scores that appears approximately Normal (Figure 3B). One consequence of recombination is that different regions of pairs of sequences will be related to each other to different degrees, and the more recombination, the stronger the homogenizing force. High levels of recombination, therefore, might “average” out the quality score across the sequence and result in the approximately Normal distribution, while low levels of recombination are expected to result in more “jagged” distributions. Overall, these analyses suggested that recombination is a major force in the generation and maintenance of variation in the *var* genes. Given the antigenic nature of the *var* genes, we suggest that recombination is an important source of novelty for the *P. falciparum var* gene repertoire. However, the evidence presented here is only circumstantial, and the extreme diversity of the sequences makes application of existing population genetic approaches to detecting recombination problematic. We are currently developing novel population genetic approaches to learn about genetic exchange among the *var* gene sequences.

### *Var* Gene Groups

To determine whether previously observed *var* gene structural groups found in the 3D7 genome [24,25] were represented within the Amele sample, we used a phylogenetic approach to identify *var* gene groupings among translated DBLα types obtained from the Amele population. This was done by observing the Amele types that clustered with 3D7 DBLα sequences upon which the groupings were partly based [24,25]. We observed that Amele DBLα types clustered significantly with all six groups identified for the DBLαCIDRα region of *var* genes [25] (Figure S7). Therefore, sequences with high levels of similarity to the DBLα domains of *var* gene structural groups previously defined in the 3D7 genome [25] were present in the Amele population. It should be noted that the structural groupings were based upon the DBLα plus CIDRα head-structure sequences [25], whereas we compared only DBLα sequences; therefore, the only group that formed a significant cluster was group A (Figures 3A and S7). Recently, an alternative grouping of *var* genes has been published based on the number of cysteine residues and short amino acid motifs within the DBLα domain [31]. We found all six of these alternative groups within the Amele population (Table S2). Therefore, the degenerate PCR primers amplified a broad range of DBLα sequences from this natural population. These results demonstrate that the functional specialization and restricted evolution of *var* gene groups suggested from analysis of a single genome [24] and African clinical isolates [31] were also observed in a random sample of a natural population of P. falciparum from PNG.

Serological analyses have predicted that the *var* genes expressed during severe disease are conserved among clinical parasite isolates [38]. Recent investigations have shown group A *var* genes to be associated with the rosetting phenotype and severe disease [31,39–41]. Although the most conserved (i.e., high prevalence) *var* genes in the Amele population were not restricted to a particular *var* gene group, at least four group A *var* genes were found in more than 20% of isolates (Table S2). These are clearly of interest as candidate vaccine targets.

The *var1csa* gene, previously found to have a global distribution [42–44], was detected in our dataset. Our analyses confirmed that *var1csa* had a high prevalence globally, but this gene was rare among Amele isolates (Table S2). This further demonstrates geographic differences among the two populations.

## Discussion

While *var* gene sequences have been extensively explored in terms of conserved features associated with adhesion [24–26,31,43,45–47], we have examined the primary sequence diversity because of its role in evasion of host immunity and consequent P. falciparum transmission. We present a population genomic framework to randomly sample the diversity of *var* genes by sequencing the ubiquitous marker, DBLα, in natural P. falciparum populations. The framework can be considered a molecular epidemiological tool for malaria with the specific application to diverse multigene families. It defines the diversity and distribution of *var* genes in natural populations. The framework could also be applicable to other diverse systems such as additional *var* gene domains (e.g., DBLα, CIDR); other multigene families encoding putative malaria surface antigens (e.g., *vir* genes in Plasmodium vivax); and the immune evasion genes of other pathogens (e.g., variant surface glycoprotein *[vsg]* genes of Trypanosoma brucei). Importantly, the cumulative diversity curve addressed the issue of incomplete sampling of individual repertoires by defining the number of isolates that were needed to see the majority of diversity in a local population (i.e., 30 isolates for Amele). If multiple infections are prevalent, which is true for many malaria populations [48–50], the extent of *var* gene diversity for the population could still be defined by plotting the cumulative curve.

Analysis of *var* gene diversity on a global and local scale using the above-described framework showed very different patterns of diversity. We were unable to sample all of the diversity in the global population even though we compared over 1,000 DBLα sequences. The global range of *var* gene diversity is likely to be so large that it would take an enormous number of isolates to observe its full extent. When *var* genes were sampled from the local population of Amele, a region less than 10 km in diameter in Madang Province, PNG, the majority of *var* gene diversity was defined. Whether this is a representation of the *var* gene diversity of Madang, or PNG in general, remains to be determined by further spatial sampling within PNG. The variation between populations indicates important differences in their evolutionary history. For example, the presence of high frequency types in Amele might indicate the effect of selective sweeps in parts of the genome that led to the fixation of specific *var* types. In addition, reduced population size, population bottlenecks, and lower levels of multiple infection may all potentially have played a role in reducing *var* gene repertoire diversity within PNG. Unfortunately, it is only by obtaining full genomic sequences for multiple isolates for each locality that the different hypotheses can be compared. Nevertheless, our findings have important implications for the understanding of local immunity in different geographic localities.

Analysis of relationships among the DBLα types found in Amele showed that *P. falciparum* has unprecedented levels of genetic diversity of a major surface antigen on a small geographic scale. This finding is particularly striking when compared to data on other prevalent human pathogens sampled locally and globally. The linear phylogenies of *ha* and *env* genes of Influenza A and HIV, respectively, are highly structured such that ancestral sequences can be traced even among isolates from distant locations [1,2], whereas the DBLα types from Amele were so diverse that the phylogeny showed only distant relationships. We have sequenced only a 500-bp fragment (DBLα) as a marker of *var* genes (>5 kb) in a limited number of global isolates and in one local population. Thus, we have described only the “tip of the iceberg” of total *var* gene diversity. This gives an indication of the vast archive of antigenic diversity available to P. falciparum and may explain why immunity to malaria is nonsterlizing and develops slowly.

To determine whether all *var* genes sampled are under similar selective pressures, it is important to know whether or not they are expressed during natural infection. In our dataset, the presence of pseudogenes could only be defined by frame shifts or stop codons in the DBLα domain. T. brucei has many *vsg* pseudogenes that have been proposed to be an archive of further antigenic diversity [51]. It is conceivable that P. falciparum also recombines fragments of *var* pseudogenes into functional *var* genes to increase its antigenic repertoire. For this reason, we included putative pseudogenes in the population genomic analysis of *var* genes where possible. In addition, only one or a few members of the *var* multigene family is expressed at a time [6]; therefore, sampling all possible transcripts from the population will require many times more sampling than for the present study. We therefore compared the Amele types to those found to be expressed in clinical isolates from the nearby population of Maiwara [32]. Interestingly, we found that 4.3% of the Amele DBLα types were expressed in the Maiwara isolates (Table S2). When a neighbor-joining tree was constructed, 22.4% of Amele DBLα types clustered with significant bootstrap support with the expressed sequences from Maiwara (unpublished data). This indicates that many of the *var* genes sampled in the Amele population were likely to be functional genes.

Our investigation of the sequence diversity among *var* genes in the Amele local population suggested that obtaining further types from Amele will only identify additional diverse *var* genes with presumably novel immunological properties. Previous studies have suggested that recombination occurs among *var* genes by analyzing distantly related genomes [21,22] and the progeny of a genetic cross [20]. Our results, namely the phylogenetic and pairwise alignment quality score analyses, suggested that recombination is an important factor in generating polymorphism among the *var* genes within a natural population. Ultimately, mutation must be the source of amino acid changes. However, the generation of novel combinations to which the host immune system has not previously been exposed is likely to be equally important to immune evasion. The complex nature of the sequence data requires new statistical tools to further define recombination among *var* genes in natural populations. As mentioned earlier, we are currently addressing this issue.

The restriction of *var* gene diversity in PNG, *var* genes in PNG which were predominantly unique to that location and the extremely limited overlap among global repertoires points to the existence of geographic population structure. Such structure has been observed for microsatellite [52] and single nucleotide polymorphism [53] markers. The majority of the broadly distributed *var* genes were found in Africa, but this may be an artifact because almost half of the global population (29 isolates) was from Africa giving a higher probability of finding matching *var* genes in that region. As P. falciparum originated in Africa [54], it is likely that the PNG population is a subpopulation of the ancestral population. The possibility of such geographic population structure warrants further investigation because genomes with different *var* repertoires may be introduced to other transmission systems with consequences for immune evasion and malarial disease. An alternative explanation is temporal variation among the Amele isolates collected in 1999 and the global isolates collected over a period of 20 y. However, our data also showed that certain *var* genes were maintained in the PNG population over long periods of time. Given the relative importance of *var* genes to P. falciparum survival [10–11], host immunity [12–19], and the potential utility of certain PfEMP1 subgroups [39,45,55] and domains [56–58] for malaria vaccination, it will now be a priority to observe spatial and temporal variation on small and large geographic scales using our validated molecular epidemiological framework.

## Materials and Methods

### Parasite isolates and cloned lines.

For global sampling, 25 P. falciparum isolates or cloned lines were chosen for their single distinct genotypes and diverse origins. From Africa there were three field isolates from Zimbabwe (collected by S. Mharakurwa from Sahumani Province, Zimbabwe in 1999. Ethical approval was obtained from the Medical Research Council of Zimbabwe); four field isolates from Dienga in Southeast Gabon, 50 km from Bakoumba (collected by FMN, ethical approval was obtained by the Ethics Committee for Research in Human Medicine, International Center for Medical Research, Gabon); D6 cloned from isolate Sierra Leone I/CDC from Sierra Leone, West Africa [59]; isolates K39, M24, and 3118 from Kenya (donated by W. Watkins, Wellcome Trust Research Laboratories, Kenyatta National Hospital, Nairobi, Kenya). PNG isolates were KF1776 (B. Tiwari, unpublished data), KF1916 [13], and Muz12, Muz37, Muz51, and Muz106 [60] collected from Amele, Madang Province, PNG, in 1986 and 1990, respectively. From Asia there were isolates SK44, PO18, PO19, and AMB47 from Thailand (donated by Wellcome Trust Research Laboratories, Faculty of Tropical Medicine, Mahidol University, Bangkok, Thailand); W2 cloned from Indochina III/CDC, isolated from a Lao refugee in 1980 [59]; isolate MRC20 from India (donated by H. Joshi, S. K. Subbarao, and C. R. Pillai, Malaria Research Centre, Delhi). From Latin America there was HB3 cloned from the isolate Honduras-I/CDC and isolated in Honduras, Central America, in 1980 [61]; and 7G8 cloned from IMTM22 isolated in Manaus, Brazil, in 1980 [62]. Parasites were cultured as described previously [13]. W2, 7G8, and D6 clones were propagated in the laboratory of D. F. Wirth, Harvard School of Public Health.

DBLα sequences downloaded from the GenBank database consisted of a total of 480 sequences from 34 isolates. These included 3D7 (number of DBLα sequences *(n)* = 57) from the Malaria Genome Project [34]; three isolates from India: R35 (*n* = 9), R15 (*n* = 9), and R1 (*n* = 7) [63]; four isolates from Sudan: SD101 (*n* = 2), SD105 (*n* = 14), SD106 (*n* = 2), and SD126 (*n* = 4) [22]; ten isolates from Kenya: KEN1 (*n* = 2), KEN2 (*n* = 8), KEN3 (*n* = 9), KEN4 (*n* = 6), KEN5 (*n* = 1), KEN13 (*n* = 5), KEN15 (*n* = 5), KEN16 (*n* = 6), KEN19 (*n* = 11), and KEN20 (*n* = 2) [33]; one isolate from Cape Verde: CV (*n* = 8) [33]; four isolates from the Solomon Islands: S2 (*n* = 28), S55 (*n* = 22), S99 (*n* = 25), and N72 (*n* = 15) [27]; two isolates from the Phillipines: PH1 (*n* = 26) and PH4 (*n* = 29) [27]; two isolates from Vanuatu: VAN232 (*n* = 4) and VAN230 (*n* = 3) [34]; one isolate from PNG: AN143 (*n* = 22); one isolate from Thailand: Dd2 (*n* = 22) [27]; one isolate from Africa: NF36 (*n* = 23) [27]; three isolates from Venezuela: VZ1 (*n* = 32), VZ2 (*n* = 15), and VZ3 (*n* = 14) [28]; and one isolate from Brazil: A4 (*n* = 33) [33].

Epidemiological sampling of a local population involved the selection of isolates from 1,082 field isolates collected for other studies in our laboratory [64]. This local population was called “Amele.” Field isolates were randomly collected in November/December of 1999 from residents of a cluster of six Amele villages in Madang on the north coast of PNG, where intense transmission of P. falciparum malaria occurs [65]. Daily inoculation rates range from 0.19–1.44 infective bites/person/night (68–526 per y) [66]. Amele isolates were collected as blood samples from children aged between 6 mo and 11 y, with asymptomatic infection. Ethical approval for the study was obtained from the Medical Research Advisory Committee of PNG.

### DNA extractions.

Whole genomic DNA was obtained from laboratory isolates as previously described [67]. Field isolate genomic DNA was obtained by one of two methods. Isolates from Gabon and Zimbabwe (from the global collection) were stored as whole blood spots on 3MM filter paper (Whatman, http://www.whatman.com), and DNA was extracted using the Chelex method. Field isolates from Amele were stored in Guanidine and extracted using Glassmilk (Qbiogene, http://www.qbiogene.com). Isolates or cloned lines were genotyped to confirm that they contained single, distinct genomes. This was done by analysis with 12 genome-wide microsatellite markers [68] and/or MSP2 fingerprinting [69].

### High-throughput DBLα sequencing.

The DBLα sequencing protocol is outlined in Figure S1A. DBLα repertoires were amplified with two sets of degenerate primers to homology blocks D (forward primer) and H (reverse primer) [26]. One set of primers known as “AF” and “BR” were described previously [33]. PCR conditions were 10 ng (cultured isolate) or 1 μl (field sample) genomic DNA, 2 mM dNTPs, 2.4 mM MgCl_2_, 50 pmol each primer, 2.5 Units Taq Polymerase (Promega, http://www.promega.com). Reactions were performed in a Perkin Elmer 9600 thermal cycler at 95 °C for 4 min, 30 cycles of 95 °C for 1 min, 42 °C for 1 min, 60 °C for 1 min, and a final cycle of 60 °C for 7 min. A second set of primers, known as “AB” (DBLαAB-F: AGRAGYTTYGCNGAYATHGG and DBLαAB-R: AACCAYCTYAARTAYTGNGG) were used to correct the amplification of nonspecific human sequences from field samples, which we found to be a problem with the AF/BR primers. PCR conditions were 10 ng (cultured isolate) or 1 μl (field sample) genomic DNA, 2 mM dNTPs, 3.5 mM MgCl_2_, 20 pmol each primer, 2 units AmpliTaq Gold DNA Polymerase (Applied Biosystems, http://www.appliedbiosystems.com). Reactions were performed in a Perkin Elmer 9600 thermal cycler at 95 °C for 10 min; 35 cycles of 95 °C for 30 s; 48 °C for 30 s; 60 °C for 45 s; and a final cycle at 60 °C for 7 min. PCR products of approximately 350–450 bp were cloned using the TopoTA PCR cloning kit (Invitrogen, http://www.invitrogen.com) according to the manufacturer's instructions. At least 96 colonies were picked from each cloning experiment and cultured in 1 ml of LB broth supplemented with 25 μg/ml of Kanamycin in deep-well culture plates. Plasmid DNA was extracted using the Millipore 96-well plasmid extraction kit (Millipore, http://www.millipore.com) according to the manufacturer's instructions, except for the vacuum steps that were replaced with a single centrifugation step encompassing both lysate removal and DNA binding. The bacterial lysate was added to the lysate clearing plate, which was then stacked on a plasmid plate with a waste collection plate on the bottom. Centrifuge adapters for stable stacking of plates are available from Millipore. Centrifugation for 40 min resulted in the plasmid DNA binding to the plasmid plate. This was followed by two wash cycles as for the vacuum protocol. Up to eight plates (768 clones) could be extracted in a few hours using this adaptation. Cycle sequencing was performed using the Big Dye Terminator Sequence Reaction mix (Applied Biosystems) according to the manufacturer's instructions, for both strands using M13 Universal forward and reverse primers. Sequencing reactions were run on an ABI3700 (Applied Biosystems) at the core sequencing center at the Department of Zoology, University of Oxford.

### Sequence analysis.

The primary sequence analysis protocol is outlined in Figure S1B and describes how raw sequence data was processed. Raw sequence data for each isolate was screened for vector contamination and end-trimmed where necessary using the automated functions in the DNA sequence analysis program Sequencher (Gene Codes, http://www.genecodes.com). Alignment was then performed at 96% DNA sequence identity to allow DBLα sequences from the same gene to align yet separate sequences from different genes. This limit was based on an analysis of data from two isolates: 3D7 [33] and 1776 (B. Tiwari, unpublished data) available from GenBank at the time of project design. Available sequences from these two isolates were aligned at incremented percentage similarity until they no longer aligned to each other, giving a value of 96%. If duplicate genes are present and diverged only within the 96%–100% identity range, these will be sampled as one *var* gene. Hence, the number of DBLα sequences within an isolate may also be a measure of within-isolate diversity if each isolate is sampled equally. Note that these numbers may also be normalized for sequence input by dividing by the number of reads. To determine whether this was a possibility, the 59 3D7 gene sequences were aligned at 96% identity. A total of 54 types remained after alignment. This showed that there was redundancy within this genome, and thus there may be redundancy within other genomes. After alignment as described, the resulting consensus DBLα sequences were considered a sample of the *var* gene repertoire. Multiple sequence reads for each consensus sequence reduced the possibility of PCR, cloning, and sequencing artifacts.

For definition of types, comparison among different *var* gene repertoires was performed. Again a 96% identity cut-off was used to align matching consensus DBLα sequences among repertoires. The number of unique DBLα sequences (types) was then defined for the population (i.e., a group of isolates). *Var* genotypes were then defined based on the range of DBLα types (Figure S1B).

To summarize the relatedness between the *var* gene repertoires from two isolates, we used a simple statistic PTS. If isolate A has a repertoire of *n_A_* distinct types and isolate B has a repertoire of *n_B_* distinct types, and a total of *n_AB_* types are shared by the two isolates, we define:

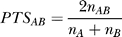



Because the isolate repertoires are not fully sampled, estimates of PTS from empirical data are likely to be downward biased. However**,** as long as there are not systematic differences between the number of reads obtained for isolates from different geographic locations the sample estimates of PTS are still useful statistics on which to base a comparative analysis of diversity. Note that if each isolate had a repertoire of exactly one *var* gene, sample PTS is equivalent to expected homozygosity, and the apportionment of PTS within and between populations would be equivalent to measuring Wright's fixation indices. PTS can therefore be thought of as a simple extension of F_ST_ to the case where multiple gene family members of unknown allelic status have been collected.

DBLα types were translated to amino acid sequences using the program Transeq from the EMBOSS toolkit at the European Bioinformatics Institute (http://www.ebi.ac.uk). Translations that contained two or more stop codons were not analyzed further as this may indicate a frame shift and may artificially result in a lack of homology to other DBLα protein sequences.

Phylogenetic analysis of DBLα types was done by constructing multiple alignments of translated DBLα types using stand-alone Clustal W downloaded from the European Bioinformatics Institute Website (http://www.ebi.ac.uk) [70]. Multiple alignments were then imported into MEGA version 3.0 [71] where neighbor-joining trees were constructed using the p-distance model to define pairwise distances between sequences. Primer sequences were removed for the phylogenetic analysis.

Pairwise alignments of “good” protein sequences (described above) were constructed using the GCG (Genetics Computer Group, http://www.accelrys.com/products/gcg) program “BestFit.” BestFit makes an optimal alignment of the best segment of similarity between two sequences using a Smith and Waterman local homology algorithm. Percent identity and percent similarity are indicators of the quality of the alignment. For scoring similarities among protein sequences, BestFit uses the blosum62 comparison matrix [72]. We also examined quality scores because percent identity or similarity scores ignore the length of the alignment. Quality scores for pairwise alignments are related to the degree of similarity but also account for length of the alignment and gaps. These will more accurately quantify the relationship among potentially recombined sequences. For example, a mosaic sequence may have 50% similarity to each of its donors across the entire length of the alignment. This would be considered equivalent to the similarity among a pair of sequences with 50% differences spread across the length of the alignment. However, the pairing of a mosaic sequence with its donor will have a higher quality score because it will score 100% similarity for half of the alignment. The calculation of pairwise quality scores was done using the following formula:

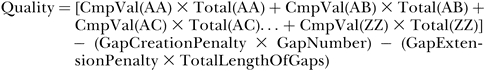



Where: “CmpVal” is the comparison value for an amino acid pair based on an amino acid comparison table (PAM matrix) and “Total” is the total number of matches for that pair in the alignment. Therefore, each match in the alignment adds points to the quality score and mismatches and gaps subtract from it.

## Supporting Information

Figure S1 A Sampling Framework for the *var* Multigene Family (A) High-throughput sequencing and (B) population genomic analysis of *var* gene sequences.
(47 KB PPT)

Figure S2 Sampling Results for Clone 3D7 and HB3 Distribution of DBLα sequences among reads using (A) 3D7 with AFBR primers [2]; (B) 3D7 with AB primers; (C) HB3 with AFBR primers [33]; or (D) HB3 with AB primers (see Materials and Methods). Bars are colored to represent distinct DBLαCIDRα groups as per Lavsten et al. [25]. Asterisks show sequences that were degenerate or recombined in our data compared to the genome sequence.
(328 KB PPT)

Figure S3 Sampling Efficiency The number of sequences obtained for each repertoire was plotted against the number of sequence reads (both forward and reverse) for global and Amele populations. Each point on the plot represents the data obtained for one PCR reaction after cloning. Up to two microtitre plates (284 sequence reads) of clones were sequenced for some isolates and lines, therefore a few points on the plot have more than 192 sequence reads. The variable number of sequence reads shown was partially due to failed sequencing reactions or erroneous amplification of non-DBLα sequences, which were discarded. Each *var* repertoire size (number of DBLα sequences obtained) varied as a result of this and other variables, such as actual repertoire size [27], PCR amplification, and DNA quantity in the field isolates.
(52 KB PPT)

Figure S4 Analysis of Primer Bias Scatterplot of the relationship between the average number of reads contributing to a type and the frequency of that type in the population. Extreme outliers can be seen above the dashed line. Arrows denote high frequency outliers.
(30 KB JPG)

Figure S5 Raw Data for PTS Matrix of the results for pairwise comparisons of *var* gene (DBLα) repertoires showing the absolute number of DBLα sequences found to be shared among a pair of isolates in the upper right hand side of the matrix; and the PTS statistic for the pair in the lower left hand side of the matrix. Self-self values are shaded in yellow.
(87 KB XLS)

Figure S6 Multiple Alignment of Amele DBLα Types Amino acid sequences for a total of 177 “good” DBLα types, defined by the absence of frame shifts and aligned using Clustal X version 1.83 [70] using the parameters: gap opening penalty = 5; gap extension penalty = 0.05.
(55 KB PDF)

Figure S7 Prediction of *var* Gene Structural Groups within the Amele Population A multiple alignment of the translated DBLα types was constructed in Clustal W [70] with parameters set at gap opening = 5 and gap extension = 0.05, for all “good” DBLα types in the Amele population (*n* = 177) with DBLα amino acid sequences from the 3D7 *var* genes [34]. The neighbor-joining tree was constructed from this alignment in MEGA version 3.0 [71] using the p-distance algorithm, pairwise alignment, and 1,000 bootstrapping repetitions. The consensus tree was condensed to show only clusters with >50% bootstrap support. Primer sequences were removed prior to drawing the tree. A key is shown to indicate the specific DBLαCIDRα sequence groupings (A–E and X) [25].
(942 KB PPT)

Table S1 GenBank Accession Numbers for New Sequences (95 KB XLS)

Table S2 Properties of Amele DBLα Types (343 KB DOC)

Text S1 Validation of a Sampling Framework for the *var* Multigene Family (55 KB DOC)

Text S2 Primer Bias (33 KB DOC)

## Abbreviations

CIDR: cysteine interdomain rich region
DBL: Duffy binding like
PfEMP1: *Plasmodium falciparum* erythrocyte membrane protein 1
PNG: Papua New Guinea
PTS: pairwise type sharing
