# Correction: Population Genomics of the Immune Evasion *(var)* Genes of Plasmodium falciparum

**DOI:** 10.1371/journal.ppat.0030070

**Published:** 2007-05-25

**Authors:** Alyssa E Barry, Aleksandra Leliwa-Sytek, Livingston Tavul, Heather Imrie, Florence Migot-Nabias, Stuart M Brown, Gil McVean, Karen P Day

In *PLoS Pathogens*, volume 3, issue 3: doi: 10.1371/journal.ppat.0030034

The seventh author's name was incorrectly listed as Gilean A. V. McVean. The correct name is Gil McVean.

