# Supplementary material for: Population Genomics of the Immune Evasion (var) Genes of Plasmodium falciparum
Source: PLoS Pathog. 2007 Mar 16;3(3):e34. doi: 10.1371/journal.ppat.0030034 (PMC1828697; doi:10.1371/journal.ppat.0030034)
Supplement: Supplementary file 1 [file ppat.0030034.sg006.pdf]

Barry *et al*. Figure S6

```
0003    -RSFADIGDIIRGKDLYLGNKK-QNQT----DR-----EKERLQQNLKEIFKKIHEKLEQ
0162    -RSFADIGDIVRGKDLFYGNP--QEKE----QR-----EK--LDEKLKEIFKKIIHEKLEQ
0163    -RSFADIGDIVRGKDLFYGNP--QEKE----QR-----EK--LDEKLKEIFKKIHDNLVE
0007    ARSFADIGDIVRGKDLFYGNP--QEKE----QR-----EK--LDEKLKEIFKKIHDNLVE
0139    -RSFGDIGDIIRGKDLYR-----RDN-----KK-----DK--LENNLKTIFKKIHDNLVE
0044    ARSFADIGDIVRGKDLFYGNP--QEKE----QR-----EK--LDEKLKEIFKKIHDNLVE
0002    -RSFADIGDIIRGKDLYLGN---N------KEK-----EN--LEKNLRQIFKKIYDKLEN
0146    -RSFADIGDIIPRKRSCISV---IP-----KEK-----EN--LEKNLSQIFKKIYDKLEN
0155    -RSFADIGDIIRGKDLYLGN---N------KEK-----EN--LEKNLRQIFKKIYDKLEN
0110    -RSFADIGDIIRGKDLYLGN---N------KEK-----EN--LEKNLRQIFKKIYDKLEN
1619    ARSFADIGDIVRGKDLYLGN---N------KEK-----EN--LEKNLRQIFKKIYDKLDD
0077    ARSFADIGDIVRGKDLYLGN---N------KEK-----KE--LEENLKKIFKEIYDKLDG
0200    ARSFADIGDIVRGKDLYIGN---RKE----REK-----EQ--LQQNLKNIFAKIYEELKN
0006    -RSFADIGDIIRGKDLYRRD---NK-------K-----DK--LENNLKTIFKQIHEKLTD
0156    -RSFADMGDIIRGKDLYLGN---NKE------K-----EN--LEKNLRQIFKKIYDKLTD
0028    -RSFADIGDIIRGKDLYRRD---NK-------K-----DK--LENNLKTIFKQIHEKLTD
0178    -RSFADIGDIIRGKDLYRGN---RKKNETKTER-----EK--LEQKLKEIFKKIYDDVTS
0033    -RSFADIGDMVRGRDLYRG----NKKEK--KQR-----DD--LEENLKKIFGDIYNEVMR
0100    -RSFADIGDIIRGKDLYRG----NN------GK-----DK--LEENLKTIFGKIH-EGLK
0106    -RSFADIGDIIRGKDLYRGN---NGKD-----------K--LRDNLKKIFKEIHDKLDK
0204    ARSFADIGDIIRGKDLFRGY---DQKDR--EQK-----KK--IQDNLKEIFKKIHDDVMK
0138    -RSFAGIGDIVRGKDLFRG----NKKEN--KQR-----DR--LEENLKTIFKQIHDDVTS
0001    ARSFADIGDIIRGRDLYGGN---NK------RR-----KQ--LDDNLKTIFTQIYEELLR
1605    ARSFADIGDIIRGRDLYSGN---KKE---KKQR-----EQ--LDDKLKKIFTQIYNDV--
0012    ARSFADIGDIVRGRDLYGGN---KKK------------EK--LEKNFKKYFQQIYNDVTR
0074    ARSFADIGDIVRGRDLYHGK---KKRGQTETER-----DK--LEENFKKYFQQIHSEVTR
1620    ARSFADIGDIVRGRDLYGGN---KKK------------EK--LEENFKKYFQQIHEELKR
0014    ARSFADIGDIIRGKDLYGRD---NKK----DKT-----DK--LQEQLKKYFNNIYDNL--
1608    ARSFADIGDIVRGRDLYRRD---NKK----DKT-----DK--LQEQLKKYFQKIYEGLTS
0073    ARSFADIGDIIRGKDLYGRN---NRK----RK-------Q--LEENLQKYFENIYYGLS-
0060    ARSFADIGDIVRGRDLYLGN---RKKKQTKTER-----EK--LDENLKRIFGKIHGGLD-
0101    ARSFADIGDIIRGRDLYIRN---KKK------------DK--LEENLQKIFAKIHGNLK-
0066    ARSFADIGDIIRGKDLYRRD---SRT------------DK--LEENLRKIFGIIYGKLK-
0191    ARSFADIGDIVRGRDLYRGD---NR------EK-----TK--LEKNLKTIFEKIYNNLN-
1617    ARSFADIGDIVRGKDLYLGK---KKRGQ--TER-----DK--LESNLKKIFGKIHEGLS-
1621    ARSFADIGDIVRGRDLFRGD---NR------EK-----KK--LEKNLQNIFKKIQEKL--
0058    ARSFADIGDIVRGKDLYLGD---NG-------K-----DR--LEENLRKIFKEIHGGLSR
0130    -RSFADIGDIVRGKDLYLGD---NG-------K-----DR--LEENLKRIFKEIYDKLD-
0229    ARSFADIGDIIRGKDLYRGN---NG-------K-----DK---QDNLKKIFKEIYDKLN-
0075    ARSFADIGDIIRGKDLYRRD---NG-------K-----DR--LEENLKTIFKKIYDNLME
0132    -RSFADIGDIIRGKDLYIRN---KK-------K-----DK--LQEQLKKYFQIIYDKL--
0181    -RSFADIGDIVRGKDLFIGN---NK-------R-----DK--LEKQLQKYFKQIYDKLDG
0187    -RSFADTGDIIRGKDLYIGN---KKE------R-----AR--LEENLKKIFKKIYEELLI
1606    ARSFADIGDIIRGKDLYLGNPQEKEK------R-----EK--LDDNLRKIFGNIYDKLLE
1622    ARSFADIGDIIRGKDLYLGNPQEKEK------R-----EK--LDDNLRKIFGNIYDKLLE
1611    ARSFADIGDIVRGKDIFIGN----KK------K-----DK--LDENLKRIFGKIYEKLD-
1887    ARSFADIGDIVRGRDLYGRD----NK------K-----DK--LQEQLKQYFNNIYDNLEG
0051    ARSFADIGDIIRGRDLYSG----NKKE----KKQR---EQ--LDDKLKEIFENIQKEN--
1613    ARSFADIGDIIRGRDLYSG----NKKE----KKQ----EQ--LDDKLKEIFENIQKEN--
0126    -RSFADIGDIIRGRDLYLG----NKK-----KKN-----Q--LEERLKTMFENI-KEN--
0004    ARSFADIGDIVRGRDIFRG----NDEK----KKR----DE--LDDKLKDIFQNIKKEN--
0010    ARSFADIGDIIRGKDLYLG----NPQE----KEKR---KQ--LDKKLKEIFENIK-KS--
0067    ARSFADIGDIIRGKDLFYG----NPQE----KEKR---KQ--LDDNLKTIFGKIKGNN--
0109    -RSFADIGDIIRGRDLYSG----NSKE----KNRR---KK--LEDNLIEIFKMIQGKN--
0153    -RSFADIGDIIRGRDLYRG----NTKE----KKKR---DE--LEKNLEIIFQNIQNKN--
0078    ARSFADIGDIVRGRDLYRG----NKKE----NKQR---EK--LEENLRKIFENIYEGLSN
1616    ARSFADIGDIVRGRDLYRGG---NTKE----KEKR---KQ--LEENLRKIFENIYEEL--
0017    ARSFADIGDIIRGRDLYRG----NSKE----K------DY--LQDKLKKYFQKIYDNLN-
0068    ARSFADIGDIVRGRDLYSG----NKKE----KKQ---RED--LENKLKEVFGKIHSEVMK
0021    ARSFADIGDIVRGRDLFYG----NPQE----KEK---RDE--LESKLKEIFGKIHGGLSK
0102    ARSFADIGDIVRGRDLFYG----NPQE----KEK---REK--LDENLRKIFKKIYDNLN-
0098    ARSFADIGDIVRGKDLYLG----DKG-----NDK--------LEKNLKRIFGKIHGGLST
0228    ARSFADIGDIIRGKDLYLG----NKKK----NEKDREKEK--LENKLKEYFEKIYNSLES
0064    -RSFADIGDIVRGRDLYLG----KKKKKQNGKETETERDQ--LEEKLQSFFKNIHDKLDD
1610    ARSFADIGDIVRGKDLYLG----KKKK-QYGKETETERDQ--LENKLKEIFGKIHGNLRN
0039    ARSFADIGDIVRGKDLYLG----KKKKKQNGKKTETERDQ--LESKLKDIFAKIHSEVTN
0085    ARSFADIGDIVRGKDLYLG----KKKK---KTETERERDQ--LEQKLKGIFKKIYEGLTG
0016    ARSFADIGDIVRGKDLYLGY---NRKE----KAQK---EK--LENKLKEYFENIHDKLE-
0174    ARSFADIGDIIRGKDLYLGY---NRKE----KAQK---EK--LEQNLKKFFQNIDEKLP-
```

```
0024            -RSFADIGDIVRGRDLYLGN---TQEK----KQRK---Q---LESKLKEIFGKIHEEV--
0168            ARSFADIGDIVRGIDLYGGN---N-------KRRK---Q---LDDKLKEIFKKIHENLG-
0046            -RSFADIGDIIRGRD-YGGS---KKEK----EKRE---Q---LDDKLKDIFKKIHGG---
0083            ARSFADIGDIIRGKDLFYGN---PQEK----EKRD---E---LENKLKDIFAKIHNGLD-
0061            -RSFADIGDIVRGKDLYSGN---KKEN----KQRE---K---LDKKLKDIFGKIHGN---
0043            ARSFADIGDIVRGKDLYLG----DKGE----KK------K--LEEKLKQYFKNI-EELKN
0177            -RSFADMGDIVRGRDLYLGY---DDKE----KDRR---DK--LEKSLKTIFGNIYEELKN
1609            ARSFADIGDIVRGKDLYLG----DKGE----KK---------LEEKLKTIFKQIYDKLTH
0202            ARSFADIGDIVRGRDLFYG----NTYE----SARR---EQ--LEKNLQNIFKQIHDKLVK
0056            ARSFADIGDIIRGKDLFIGY---NQKD----RKEK---AK--LQENLRKIFKDIHNNLQ-
0089            ARSFADIGDIVRGKDLFIGY---NQKD----RKEK---EQ--LQNKLKYIFKKIHEKL--
0059            ARSFADIGDIIRGKDLYLGD---KKKD----QQEK---VR--LEENLKKIFGHIYEELLI
0081            ARSFADIGDIIRGKDLYLGG---RGRD----Q----------LESNLRKIFAKIHDKLY-
0013            ARSFADIGDIVRGRDLYLG----NPQEIK--QR-----QQ--LENNLKTIFGKIYEKLN-
0193            ARSFADIGDIVRGRDLYSG----NDEEKK--QR-----KQ--LDKKLKVIFGKIYKDLTK
0203            ARSFADIGDIVRGKDLYLGY---DDKEKK--RR-----EK--LEQKLKEIFAKIHEELKN
0063            -RSFADIGDIIRGKDLY-G------GNNK--RR-----QQ--LEENLKTIFGHIYEELKK
0198            -RSFADIGDIIRGKDLY-G------GNNK--RR-----QQ--LEENLKTIFGHIYEELKK
0048            ARSFADIGDIIRGKDLY-R------GVN---GN-----DK--LEDNLKKIFGIIYEELKK
1676            ARSFADIGDIVRGRDLYRG------GNNK--RR-----QQ--LDDKLKKIFGKIHDEVTR
0015            ARSFADIGDIIRGKDLYRG----NK-EKD--RR-----RK--LEENLKTIFGDIYKELSR
0149            -RSFADTGDIVRGRDLYRG----NKQEKE--KR-----KK--LQQNLKTIFGKIHEGLSE
0127            -RSFADIGDIVRGRDLYSG----NKK------K-----ER--LDENLKTIFKNIYEKLLE
0148            -RSFADIGDIVRGRDLYRG----NNKERN--RR-----DQ--LESKLKDIFKNIYKDVTS
0042            ARSFADIGDIIRGKDLYSR----DNR------K-----DK--LQTKLKEYFKKIYYGLTD
0023            -RSFADIGDIIRGRDLYSG----NKVK-----K-----KK--LDDSLKTIFGKIYEGLTG
0208            ARSFADIGDIVRGKDLYSG----NNKEKN--RR-----QQ--LEQKLKDIFKKIYEELKD
0020            ARSFADIGDIVRGKDLYRGY---DDEEKK--KR-----ED--LEKNLKEIFKEIHGGLT-
0091            ARSFADIGDIIRGKDLYVG----NRKEKE--K------EK--LQKNLKNIFAKIHSGLT-
0170            ARSFADIGDIIRGKDLYLG----DIKKKQ--NGKKTEREK--LEENLKRIFGKIHEDVTN
0131            -RSFADIGDIVRGRDLFYG----NDEEKK--QR-----DK--LDENLKEIFKKIHSEVTS
0169            ARSFADIGDIVRGKDPFYG----NTYEST--QR-----EK--LEKNLKDIFGKIHDDVTS
1764            ARSFADIGDIVRGKDPFYG----NTHESA--QR-----KV--LDKKLKEVFGKIHDDVTS
0215            ARSFADIGDIIRGKDLFYG----NTQESA--QG-----KK--LDDKLKEIFKEIHDEVTK
0226            ARSFADIGDIVRGKDPFYG----NTYESA--RR-----EK--LEENFKKYFQQIHDDVTK
0221            -RSFADIGDIVRGKDLYLG----NPQESA--RR-----DK--LENKLKEIFKEIHSEVTK
0019            ARSFADIGDIVRGRDLY----LRDKGK---RDK---------LEEKLKTIFGIIYGKLKN
0223            ARSFADIGDIVRGRDLYGGS-KKEKDR---RDQ---------LESKLKDIFGKIYQELTT
0227            ARSFADIGDIVRGRDLY----RGNDEE---KKK---------LEKKLKKIFGIIYDNLVQ
0040            ARSFADIGDIIRGKDLYRGNNGKDKAQ---KLK---------LQDNLKEIFQKIQ-----
0201            ARSFADIGGIVRGRDLYR----RDKGE---ETK---------LENKLKDIFKMIQ-----
0071            ARSFADIGDIVRGKDLYR----RDKGE---ETK---------LEKNLKEIFRRIQ-----
0052            ARSFADIGDIVRGRDLYR----RDKGE---ETK---------LEKNLKTIFGKIHK---E
0072            ARSFADIGDIVRGKDLFL----GNSVE---SAQR----II--LEKNLKGVFGNIYNELTT
0079            ARSFADIGDIIRGRDLYR----RDKGE---ETK---------LEKNLKEIFKNIYNELTT
0031            -RSFADIGDIVRGRDLYLG----NPQE---SAQR----KQ--LDENLKTIFKNIYEKLLE
0179            -RSFADIGDIIRGKDLYLG----NDKE---KDQR----KV--LDENLKTIFKNIYEKLLQ
0134            -RSFADIGDIIRGRDLFYG----NTQE---STQR----KQ--LDDKLKDIFGNIYEGL-K
0147            -RSFADIGDIVRGKDLFLG----NTYE---SAQR----KV--LDEKLKEIFKNIR----K
0133            -RSFADIGDIIRGKDLYLG----NPQE---IAQR----KE--LDKKLKDIFAKIYEE-LK
0137            -RSFADIGDIVRGKDLFLG----NSVE---SAQR----KV--LDENLKKIFKQIHEK-LD
0057            ARSFADIGDIVRGKDLFLG----NDKE---KEKR----KQ--LDENLKTIFKNIYEKLLE
0032            -RSFADIGDIVRGRDLYR-----RDSR---TD-------K--LEENLKVIFGNIYKELTS
0107            -RSFADIGDIVRGKDLFLG----NDKE---KDQR----KK--LEKNLKTIFGNIYKELK-
0008            -RSFADIGDIIRGKDLFIGYNEKDRKE-----K-----EK--VQKNLKKIFRKIYEELKG
0196            -RSFADIGDIIRGKDLFIGYNEKDRKE-----K-----EK--VQKNLKKIFRKIYEELKG
0129            -RSFADIGDIIRGKDLYLGYDDKEKKR-----R-----EK--LENKLKVIFGHIYEELTT
0053            ARSFADIGDIIRGKDLFIGYNQKDREQ-----K-----KK--IQDNLKDIFGKIYKELTT
0080            ARSFADIGDIVRGRDLFIGYDKKDREQ-----K-----KK--IQDNLKEIFKKIYDEVTR
0099            ARSFADIGDIVRGKDLFLGNDE-EKKK-----R-----DE--LEENLQKIFAKIHSGLTS
0152            -RSFADSGDIVRGKDLFLGNDE-EKKK-----R-----KQ--LQQNLKNIFGKIHSDVMK
0005            -RSFADIGDIIRGKDLFLGG---PDQ-----EK-----KK--LEENLRNIFKNIHDHLT-
0026            -RSFADIGDIIRGKDHYLGN----NQ-----EK-----EK--LQENLRQIFQNIMDKNE-
0151            -RSFADIGDIIRGKDLYLGY---NKK-----EKAQK--EK--LEQNLNFFFQNIDDK-L-
0180            -RSFADIGDIIRGKDLYRGG---NTK-----EKRQR--QK--IEDNLKEIFKNIKN--T-
0054            ARSFADIADIVRGKDLFLGG---PNQ-----EK-----KL--LEEKFKKYFQTLYNN-L-
1886            ARSFADIADIVRGKDLFLGH---SK------EK-----EK--LEQNLKNLFKQLYEKLN-
1618            ARSFADIGDIVRGKDLYLGT---NE------EK-----KP--LEENLKKLFKKLYTDLK-
1612            ARSFADIGDIVRGKDLFLGH---SK------EK-----EK--LEQNLKNLFKQLYEELKK
0011            ARSFADIGDIIRGKDLYRGN---NK------EK-----DR--LEDNLKKIFKQLYEELTK
0037            ARSFADIGDIIRGKDLYLGN---KKHNEAEREK-----EK--LQRNLKYIFKKIYDNLS-
```

```
0049      ARSFADIGDIIRGKDLYRGN---KKYNETEREK-----EK--LQRNLKYIFKKIYDNLV-
0027      -RSFADIGDIIRGKDLYLGN---KKRNENLREK-----EK--LEIKLKSFFKNIYNNLT-
0034      -RSFADIGDIIRGKDLYRGD----EKK-----------EK--LELKLKSFFKNIYDDLIN
0069      ARSFADIGDIIRGRDLYRGK---RQEK-----------DY--LQEKLKNYFQKIYDDLAK
0055      ARSFADIGDIVRGRDLYRGN---NREN-----------DK--LEKKLKEYFKKIYEQLVK
0104      -RSFADIGDIIRGKDLYRGN---NGK------------DK--LEENLKTIFKKIHGGLTG
0030      -RSLADIGDIVRGRDMFRSN------------------EN--VEKGLRVVFKNIHENLRG
0160      -RSFADIGDIVRGRDMFRSN------------------EN--VEKGLRAVFKNIRENLRG
0022      -RSFADIGDIVRGRDMFKSN------------------EK--VEYGLRKLFKKIHENLRG
0171      -RSFADIGDIIRGKDLYLGN---NKE------K-----EN--LEKNLRQIFKKIYDKLEN
0041      ARSFADIGDIVRGRDMFKSN------------------ED--VEKGLKVVFKKIQRKLNV
1607      ARSFADIGDIVRGKDMFKSN------------------EK--VEYGLRKLFKKIH-DLNK
0216      ARSFADIGDIVRGRDMFKSN------------------EK--VEIGLKKVFEKINNGLKK
0082      ARSFADIGDIVRGIDMFKSN------------------DK--VEKGLREVFKNIYEGLKG
0076      ARSFADIGDIVRGKDMFKPN---DK-------------DE--VWKSLRAVFKKINDNLKE
0214      ARSFADIGDIVRGIDMFKPN---VH-------------DK--VEKGLREVFKKIHDEMEG
0047      -RSFAD-GDIVRGKDMFKRT---DN-----------------VEKGLRAVFGKIYKSLTF
0141      -RSFADIGDIVRGRDMFKRT---DK-------------DY--VENGLREVFNNIYGQLEN
0197      -RSFADIGDIVRGKDMFKSN---KN-----------------VEIGLKKVFNKIYDKLKQ
0065      -RSFADIGDIVRGRDMFKPN---P--------Q-----DK--VQEGLKNVFKNIHENFP-
1614      ARSFADIGDIVRGRDLYRGN---NG-------K-----DK--LENNLKEIFKKIYEELIK
0036      ARSFADIGDIIRGKDLFIGY---NQKDRK--EK-----EQ--LQNSLKIIFREIYNKLHN
0084      ARSFADIGDIVRGKDLFNGY---NQEDIK--EK-----AK--LQENLRKIFAKIYNNLEN
0143      -RSFADIGDIIRGKDLFRGY---NEID-E--QK-----KK--IQDNLKDIFAKIYNSLKD
0029      -RSFADIGDIIRGKDLFLGH---KQ------RK-----KE--LEENLRKIFANIYKELTS
0038      ARSFADIGDIIRGKDLFLGN---NDNDKI--KK-----EK--LQGNLEKIFKRFKA--KY
0218      ARSFADIGDIIRGRDMFLGN---NEKDMR--EK-----QK--LQSNLKKIFEKIRN--DG
0050      ARSFADIGDIIRGKDLFLGH---KQG------K-----EH--LEKRLESMFKNIQN--EN
0224      ARSFADIGDIIRGKDLYRRD---KGG------K-----TK--LEKNLEKIFQYIKE--NN
0070      ARSFADIGDIIRGKDLFLGH---QQK------K-----SV--LEASLKSMFENIK---KN
0190      ARSFADIGDIIRGKDLFLGH---KQR------K-----KY--LEESLKSMFDNIK---KN
0194      ARSFADIGDIIRGKDLYLGH---EQG------N-----NR--LEARLKTIFQNIQN--KN
0173      ARSFADIGDIVRGKDLFLGH---QQR------K-----RK--LEENLKQMFENIK---KN
0167      ARSFADIGDIIRGKDLYLGY---DQKEKE--QR-----DK--LEEKLQKIFQQIHSDVTS
1615      ARSFADIGDIIRGKDLYRG----NTKEKK--KR-----ED--LENKLKEIFAKIHSDVTS
           **:.. ..::                                   :   *  :

0003      P------------AKERYEGD---EANN--FF-KLREDWWNANRQQIWKAITCNVP----
0162      P------------AKERYEGD---EANN--FF-KLREDWWNANRQQIWKAITCNVP----
0163      KKGN--------EAKDRYKDE---PDNN--FY-QLREDWWMANRDQVWKAITCNVP----
0007      KKGN--------EAKDRYKDE---PDNN--FY-QLREDWWMANRDQVWKAITCKAD----
0139      KKGN--------EAKDRYKDE---PDNN--FY-QLREDWWMANRDQVWKAITCKAD----
0044      KKGN--------EAKDRYKDE---PDNN--FY-QLREDWWMANRDQVWKAITCKAD----
0002      E--EAK---------EYYKN--NDDTEN--YY-KLRNAWWEANRQEIWKAITCSAA----
0146      E--EAK---------EYYKN--NDDPEN--YY-KLRNAWWEANRQEIWKAITCSPA----
0155      E--EAK---------EYSKN--NDDPEN--YY-KLRNPWWEPNRQEIWKPIACSAA----
0110      E--EAK---------EYYKN--NDDTEN--YY-KLRNAWWEANRQEIWKAITCSAA----
1619      A--VIQ---------KHY----DDAKGN--YY-KLRNDWWEANRQEIWKAITCGAG----
0077      K--NGK---------KTLQERYKDDKEN--FF-QLREDWWALNRQDVWKAITCEHP----
0200      G--KTN---------GIEER-YNDDTTD--FF-KLREDWWALNRQQVWYAITCGAA----
0006      L--RAN---------DHYKD---ENGGN--FF-KLREDWWTANRATVWEAITCRYP----
0156      L--RAN---------DHYKD---ENGGN--FF-KLREDWWTANRATVWEAITCRYP----
0028      L--RAN---------DHYKD---ENGGN--FF-KLREDWWTANRATVWEAITCRYP----
0178      TSGRTNGK----TLQARYEG---DEANN--FF-KLREDWWTANRHTVWKAITCDVV----
0033      GR-NVKTN----GIEERYGN-----DPN--FF-QLREDWWTANRAKVWEALTCDAPH---
0100      ---NGKTN----GIEERYGN-----DPD--FF-QLREDWWTANRETVWKAIRCSAPR---
0106      --------------KDRYND----DTPD--FL-KLREDWWDANRHTVWKAITCGAPH---
0204      T--SGRTKGA-EELKARYKDR---KDPD--FF-KLREDWWNANRETVWKAITCGAG----
0138      T--SGKNR---LALQERYKED---PDKN--YS-KLREDWWNANRQEVWKAITCGTHD---
0001      ---TSTNVEA---LKTRYK--D--TTN---YY-ELREDWWEANRAKVWYAITCDA-----
1605      ---TKTNGE----LKRRYK--D--TDN---YY-ELREDWWDENRHTVWEAMTCDAR----
0012      TSTSGNNKDT---LQERYKE-D--PNNN--YF-QLREDWWTANRHTVWEALTCEVG----
0074      ----GSNWQA---LQKRYKG-D--KENY--FF-QLREDWWTANRATIWEALTCEAP----
1620      ----GDKTKE---AEKHYQ--D--TTN---YS-KLREDWWTANRHTVWKALTCDDR----
0014      ---EG----A---AKKHYNG----DKNN--FF-KLREDWWYANRQQVWKAITCKA-----
1608      T--NGRNGEA---AKKHYED----GAPD--FL-KLREDWWEENREKIWKAITCKA-----
0073      ---NETNAKE---LQERYKD----DTPD--FF-KLREDWWSLNRETVWKAMTCSAD----
0060      ------DK-----IKSNYDD----SEGN--FY-QLREDWWALNRQDVWKAITCSAA----
0101      ------D------ARTHYSD----TDKN--YY-QLREDWWTANRATIWEAITCEAG----
0066      ------D------AQSHYSD----TT-N--FY-QLREDWWALNRKEVWKAITCEAG----
0191      ------NPK----AKTHYEDKD--PEKN--FF-KLREDWWTANRHTVWKAITCGAY----
```

```
1617   ------TKNG---AQNYYEDKD--TDKK--FF-KLREDWWTANRHTVWKAITCGAY----
1621   ------TGD----AQTHYED----ATEN--FY-QLREDWWALNRVQVWNAMICGVE----
0058   A--NGKKGQK--PAKEYYK-DE--NGGN--YF-QLREDWWTANRATVWYAITCGAG----
0130   ----GNKG-K--EAKKHYQ-DT--K--N--YY-QLREDWWYANRKMVWYAITCGAE----
0229   ----GKNGKK--TLQKRYQ-DD--GTGN--YY-QLREDWWNNNRIMVWHAITCGAA----
0075   D--LKKDPTKSAEAQKRYKKDE--E--N--YY-QLREDWWTANRSTIWEAITCDAK----
0132   ----KDKEG----AKERYK-D---TA-N--YY-ELREDWWALNRQDVWKAITCEAG----
0181   ----KKKEE----AKARYN-DE--SG-N--YF-QLREDWWNNNRKMVWYAITCHAG----
0187   S--TSDKNGE---LQKRYN-DE--SG-N--YY-LLREDWWNINRKKVWDAITCEAQ----
1606   E--NRTNGRNGEAAKKRYEGD-----DN--YY-QLREDWWTANRETVWKAITCDAP----
1622   E--NRTNGRNGEAAKKRYEGD-----DN--YY-QLREDWWTANRETVWKAITCNVS----
1611   -------------KKDRYNGD---G-DN--YY-QLREDWWTANRATVWKAITCEAP----
1887   -------------VQNHYNGDK--N-NN--YY-QLREDWWTANRETVWEAITCDAP----
0051   --------------NSNLKSL---TDD------QIREYWWALNRQEIWKAITC----DAP
1613   --------------NSNLKSL---TDD------QIREYWWEANRKEVWKAITC----DAP
0126   --------------NGELKKL---LTE------QIREYWWALNRKEVWKALTCS---DKL
0004   --------------NSKLTNL---NDD------QIREYWWDANRHTVWEAITC----NAG
0010   --------------DTKLSTL---EDE------EIREYWWALNRLEVWKAITC----HVV
0067   --------------KSKLGQL---KLD------KVREYWWNANRAKVWEAITC----KAK
0109   --------------ESKLGAL---SLD------QVREYWWELNRETVWKAMTCS---DRL
0153   --------------ED-LKSL---SLD------EVREYWWDANRATVWKAITCD---NKL
0078   N-----------GVKARYEGD---KEN---FY-QLREDWWALNRDQVWKALTCS---DDL
1616   K-----------GAKTRYEGD---KNNN--FF-KLREDWWALNRDQVWKALTCD---DRL
0017   ------------GAQTYYNDN--DKDKN--YY-QLREDWWIANRDQVWKAMTCSDDDKKL
0068   T-----------SGRTNGTEERYGKDPD--FY-QLREDWWTVNRDQVWKAMTCSDDDKKL
0021   K-----------DAQNYYKDD---AKKN--FY-RLREDWWTANRHTVWKAITC-DEENKL
0102   ------------GAQTHYSDT---T--N--FY-QLREDWWNANRATIWEAITC-NDDNKL
0098   KN----------GAQKHYQDE---NGGN--FF-KLREDWWEANRKDVWKALTC-SD--KL
0228   S------------IKSQYDDE---AN-N--FF-QLREDWWEANRKEVWKALTC-DDDKKL
0064   S------------IKSNYN-D----APY--YY-KLREDWWTANRETVWEAMTCS---KEL
1610   G------------VKARYIGD----TTD--FF-KLREDWWNANRETVWKAMTCS---KEL
0039   GR----NGK-NREIEKRYEGD----D-N--YY-QLREDWWNANRETVWKALTCNED-KKL
0085   G------------VKDHYQ-D----TDN--YY-ELREDWWALNRDQVWKAITCK-----A
0016   -----------QRAKEHYIKD--KETGN--YY-KLREDWWTANRHTVWEALTCEVG----
0174   -----------LKAKNYYTKE--KDP-N--FL-KLREDWWTANRETVWEALTCDA-----
0024   -----------TRGKKGAQTRY-NDTDN--YF-QLREDWWALNRDQVWKAITCEA-----
0168   -----------THEKKHYE----NDTAN--YY-KLREAWWALNRQDVWKALTCDA-----
0046   -----------SKEKISHYNG---DTPY--YY-KLREDWWTANRHTVWEALTC-----KA
0083   -----------GKTLQKRYNG---DDPN--YY-KLREDWWTANRATIWEALTCDED-NKL
0061   -----------LKDARTHYS----DTTN--YY-KLREDWWTANRSTVWEAITCG----AP
0043   GK----TNG---EIEARYGNDPK-G--D--FF-QLREDWWNANRETVWEAITCSDK---L
0177   GK----TNGKNGEIETRYKGDAK-K--N--FY-QLREDWWEANRQEVWKAITCSEK---L
1609   ------------DAQTHYKKDDK-GSEN--YY-QLREDWWDANRETVWKAITCSEK---L
0202   DN----E-----KAKDRYNGDG--G--N--FF-KLREDWWDANRETVWKAITCSED---L
0056   ----------DTEAQKRYKNNDE----N--YF-QLREDWWTANRATIWEAITCDED-KKL
0089   ----------DSEAQTRYNDATG----N--FY-QLREDWWTANRATIWEAMTCSED---L
0059   ST----SG--RNGVKDHYQDENG-G--N--FF-QLREDWWTANRATIWEAITCDEE-NKL
0081   -----------NSIKSNYNDAP-----N--FF-QLREDWWTANRATIWEALTCDA-----
0013   -----GA----EA---RYGND----PE---FF-KLREDWWTANRETVWKAITCN------
0193   KKGKKGA----EELQKRYGSD----EN---YY-QLREDWWTANRETVWKAITCN------
0203   ----GKK----SALQERYTND---GPE---FF-QLREDWWNANRQEIWKAITCNDDNK--
0063   D-RKNGA----EELQKRYNGD---EANN--FF-QLREDWWTANRNDVWKAITCSADK---
0198   D-RKNGA----EELQKRYNGD---EANN--FY-QLREDWWMANRDQVWKAITCKADN---
0048   D-PTK------SELQARYNGD---GPE---FF-KLREDWWYINRKDVWKAIRCSAPK---
1676   R-KQNG-----QALQARYQDE---NGGN--FF-KLREDWWTANRNDVWKAITCDDDNK--
0015   TNGE---------IEARYK-D---AKNN--FY-QLREDWWTANRHTVWKAITCNA-W---
0149   S------------AKEHYKGD---KENY--FF-QLREDWWTANRSTVWKAITCKADT---
0127   DN-KTNGKK--LALQKRYQDE---IGGN--FF-KLRDDWWTANRETVWKAITCSADR---
0148   TNGKTNG------AEARYNDD---TG-N--YY-KLREDWWTANRATVWKAITCSEDL---
0042   QK-----------AKQHYNGD---GD-N--YY-KLREDWWTANRETVWKAMTCSDDL---
0023   GV------------KERYTND---GG-D--FF-QLREDWWTAN-ETVWKAMTCSEHL---
0208   A-------------KDRYKND---TE-N--YY-KLREDWWTANRATVWKAITCNA-----
0020   ----KNG------AQNYYKDK---NGDN--FF-KLREDWWDANRATVWKAMTCSEKL---
0091   ----KKG------AEARYKD----NDEN--YY-QLREDWWEANRAKVWEAMTCS--V---
0170   GK--KEV------LKTRYKDI---NDPE--FF-KLREDWWTANRATVWKAITCHAGE---
0131   GSNGK--------LKKRYKDD---DKKN--FY-RLREDWWTANRHTVWKAITCDAKA---
0169   NSSNKDA------LKTRYKDD---DTEN--YF-QLREDWWTANRETVWKAMTCKADT---
1764   G------------AQTRYKKD---G-GN--YF-QLREDWWTANRETVWYAITCGAGT---
0215   GRS-ASP------LQARYQGD---DNNN--YS-KLRKDWWTANRETVWKALTCDAPG---
0226   GGTNGQT------LQERYKGD---DNNN--YS-KLREDWWTANRETVWKALTCDV-----
0221   GQT----------LKTRY-GS---DAPD--FF-KLREDWWDANRATIWKAITCDAK----
```

```
0019            GKTNGQK-----SAKEYYEDK---DTDK-NYY-KLREDWWALNRQQVWKAITCNAG----
0223            -TTNGKK-----SAKEYYKD----DTDG-NYY-KLREDWWNANRQDVWKAITCNAR----
0227            ---N-KK-----DAKSRYND----DTK--NYF-QLREDWWALNRDQVWKAITCNAG----
0040            -RKNQS------KLSQLT-------LD------QVREYWWEENREKVWKAITCNA-----
0201            -GKNQS------KLSQLT-------LD------QVREYWWERNREKVWKAITCNA-----
0071            -GKYP-------KLNGLP-------LD------KVREYWWDANRLDVWKAITCDAP----
0052            VTKGSNG-----KLKARYG------SDKENYY-QLREDWWEENRETVWKAITCNA-----
0072            SGKNVD------KAKERYKG----DADNN-YF-QLREDWWEENRETVWKAITCD-V----
0079            --KN--------GTKERYN-----DTDN--YF-QLREDWWEENRETVWKAITCHVV----
0031            DNKTNG-------AEARYKDD---GG-D--FF-QLREDWWNANRKEIWKAITCHAR----
0179            DNKTSGKK---EEIERRYKGD---AKKN--FY-QLREDWWTANRHTVWKAITCDAR----
0134            NGKTNGK----NVDTLKDRYE---GDDN--YY-QLREDWWALNRDQVWKAITCEAG----
0147            N----------NYSTLNDLS----------LE-QVREYWWALNRETVWKALTCDAG----
0133            NGKN-GKN-A-EELKARYQDE---NGGN--YY-QLREDWWDANREKVWKAITCNAG----
0137            S-----------EAQKHYKDD---NGGN--YY-QLREDWWDANRETVWKAITCNAG----
0057            DNKTSGKNGA-EELQKRYKKD---EDPN--YY-KLREDWWEANRETVWKAITCDAG----
0032            TNGKNGK----KDAEERYKGD---EANN--FF-KLREDWWTENRETVWKAITCDAH----
0107            KDRKNGE----EELQKRYKKD---EDKN--FF-KLREDWWEENRQQVWKALTCDAH----
0008            AQTYYED--------K--DT----DK---NFF-QLREDWWTENRGTVWKAITCDAH----
0196            AQTYYED--------K--DT----DK---NFF-QLREDWWNANRKEVWKAITCKAN----
0129            SREKSAEA------QKRYGS----DAP--YYF-QLREDWWALNSQDVWKAITCEAK----
0053            TNG-KNGKNA-EELKARYED----ESG--NYF-QLREDWWDANRKKVWDAITCKAN----
0080            --G-KNG-----EIETRYGS----DE---NYF-QLREDWWDANRAKVWYAITCGAG----
0099            TRGKQNG-----ELQKRYKG----DAKK-NFY-QLREDWWDANRHTVWEAITCGAG----
0152            TSG-SNG-----QIEARYGS----DAP--YYY-QLREDWWTVNRDQVWKAITCKAN----
0005            DAKA----------KSYYKND-----NDRNYF-KLRNDWWELNRQQVWNAITCGAP----
0026            DLKD----------LTL---D-----QVREY-------WWALNRQDVWKAITCNAP----
0151            DPEA----------KNYYAKE-----KDPDFL-KLREDWWELNRQDIWKALTCNAP----
0180            NPRL----------TDL---------TD-D---QIREYWWKANREKVWKALICHAP----
0054            TGEA----------RDHY--N-----NDYPYFYQLREDWWNANRYDVWKAITCNA-----
1886            DPQ-----------KSSYNDD-----GTGNYY-KLRNDWWELNRQEIWKAIICQAP----
1618            DPK-----------KSSYNDD-----GTGNYF-KLRNDWWELNRQEIWKAIICQAP----
1612            NNKNEA-------IKTHYEKD-----GQ-NYY-KLRNDWWELNRQEIWKAITCDAP----
0011            NNKNEA-------IKTHYQDD-----DP-NYY-KLRNAWWEANRQEVWKAITCGA-----
0037            DPK----------ARDHYNDA-----GE-NYY-KLREDWWNANRLDVWKAITCN-D----
0049            KNKED--------ATGHYGGD------E-NYY-KLREDWWDANRQEIWKAITCSAD----
0027            EE-----------AKQTYQDN-----DE-NYY-KLREDWWDANRETVWEAITCN-A----
0034            DQG------KKDAAIKHYFDP----KGN--YY-QLREDWWDLNRHDVWKAITCSAP----
0069            DK-------NGEVAKEYYISD----APN--YY-RLREDWWDLNRHDVWKAITCDAP----
0055            KY--------KEKAETDYKDD----TKN--YS-KLREDWWELNREKVWNAITCYAH----
0104            GV--------TDR----YKD-----TVN--YY-QLREDWWEANRETIWRALTCDAP----
0030            -------------PAKSHYED---GDKSGNYY-KLREHWWIVNRKQLWEAITCIAP----
0160            -------------PAKNYYAD---DDGSGNYY-KLREHWWIVNRKQLWEAITCIAP----
0022            -------------PAKSHYED---GDKSGNYY-KLREHWWIVNRKQLWEAITCIAP----
0171            E------------EAKEYYKN---NDDTENYY-KLREHWWIVNRKQLWEAITCIAP----
0041            -------------AAKS-YYN---ADEKGNYY-KLREDWWTVNRDQVWKAITCKAP----
1607            -------------SKIN-DYD---GDGP-EYY-KLREAWWTANRDQVWKALTCSAP----
0216            -------------IGIH-DYD----DISGNYY-KLRDAWWTVNRNQVWEAITYKAP----
0082            -------------EGVKVHYK---ENKDGNYY-KLREDWWTANRDKVWKALTCNAP----
0076            -------------KGIH-DYD----DKYGNYF-KLREAWWNANRDKVWEAITCNAP----
0214            -------------EVKN-YYN---PDGSGNYY-KLREAWWDVNRNKVWEAITCGAL----
0047            -------------PAKNYYAD---HDKSGNYY-KLREDWWTVNRDQVWKALTCTAP----
0141            -------------NAKAYYSD---KDKSGNYY-KLREAWWNANRDQVWRAITCKAP----
0197            -------------PAKSYY-N---ADGEGNFY-KLREDWWTANRDQVWKAITCEAP----
0065            ---------E---PLKRHYAD---HDKSGNYY-KLREDWWTANRDQVWKALTCSAD----
1614            NGSN----GK---EAQEYYKK---DDKDPNYY-KLREDWWTANRDQVWKALTCSAD----
0036            TE-----------AKRHYNDE---DDNEGNYF-QLREDWWDANRKEVWKAITCSAG----
0084            TE-----------TKKRYNEE---DDNEENYY-KLREDWWALNRQEVWKAITCGAG----
0143            KKG----------AETRYG-------SDENYF-QLREDWWDANRKEVWTAITCDAG----
0029            TSDKNGKNGQ---IEARYG------SDTKNYF-QLREDWWALNRDQVWKAITCAAK----
0038            ED----------------------INNLPID-DIREYWWALNRNDVWKAITCSAP----
0218            RT----------------------PKNVPIG-QVREYWWALNREDVWKALTCSAD----
0050            KD----------------------LTTIPLE-KFREYWWALNRKEVWKAITCDAG----
0224            KSS---------------------LESLTDE-EIREYWWALNRDQVWKAITCNAP----
0070            NEK---------------------LNSLQNE-QVREYWWALNRDQVWKAITCAAD----
0190            NEK---------------------LNSLENE-QVREYWWEANRNQVWKAITCGAT----
0194            KSP---------------------LDKLSVD-KVREYWWALNRDQVWNAITCGAG----
0173            NDK---------------------LDKISIE-QVREYWWNANRDQVWEAITCHAA----
0167            T--N-GKLK------TRYED-----NDE-NYF-QLREDWWNANRKEVWKAITCKAP----
1615            TSGNKGKMNG---AEARYKD-----DGSGNFY-RLREDWWEANRETVWKALTCNAP----
                                                      **  *   :* .:
```

```
0003          -EDAKYLEKK-DGRAYG---CTHTS---------CHCA-------VGD----------V
0162          -EDAKYLEKK-DGRAYG---CTHTS---------CHCA-------VGD----------V
0163          -EDAKYLEKK-DGRAYG---CTHTS---------CHCA-------VGD----------V
0007          -NSNRYFRQT-CGSGEK---GSATTG-------KCRCSDKPKSGKPGDVS--------IV
0139          -NSNRYFRQT-CGSGEK---GSATTG-------KCRCSDKPKSGKPGDVS--------IV
0044          -NSNRYFRQT-CGSGNN---PTLARD-------KCRCKNK-K--VTNDTD--------QV
0002          --GGTYFRKT-ACSSY------YPTG------DKCRCT-----------------THDV
0146          --GGTYFRKT-ACSSY------YPTG------DKCRCT-----------------THDV
0155          --GGTYFRKT-ACSSY------YLPG------DKCRCA-----------------THDV
0110          --GGTYFRQT-CGNND------NWTE------EDCRCAG----------------NIRV
1619          --GSQYFRTT-ACSRN------YQTG------EDCRCR-----------------TNDV
0077          --GGTYFRKR-ACSSY------YPTG------EDCRCK-----------------TNYV
0200          --GGTYFRGT-CGKND------TWTR------EDCRCDG----------------SNVV
0006          --GGRYFRQT-CGNND------NWTE------EDCRCAG----------------NIRV
0156          --GGRYFRQT-CGNND------NWTE------EDCRCAG----------------NIRV
0028          --GGRYFRKT-ACSSY------YPTG------DKCRCT-----------------THDV
0178          --SGTYFRPT-CGDSDGR--SEYQTQ------KQCRCEG----------------ANVV
0033          --NAQYFRGT-CG-SSAK--TATRTP------SQCRCEGAKSA-----------NVDQV
0100          --DADYFIKT-VC-SGGK--TPTQG--------KCRCIDFS-----------------V
0106          --DSKYFRQT-CG--NGK--GPSQAK------NKCRCSDKP-------------NTDQV
0204          --GGTYFRAT-CGGDNEK--NATLAS------NKCRCDDKTSGAKDG--------DVNIV
0138          --GASYFRAT-CDINNEK--GPSVAQ------KQCRCDDKPKGGKAGKGSG----DVNIV
0001          QG-FNYFRPT-C--NG----G-KRT------EGYCRCDGD-----------------QV
1605          EG-DKYFRQT-A--CA----G-TGT------QGKCRCSDN-----------------QV
0012          S--GTYFRPT-C--NG----G-ERT------EGYCRCNDDKPGHDRP---N------TDP
0074          EH-ASYFRAT-CSMNGS---G-AQA------NDKCRCKKNDGTHD-----S------DQV
1620          LAGASYFRAT-CD-TGK---GPSQA------HDKCRCKDENGKSE-----T------DQV
0014          EG--AYFHAT-CDSGDEK--GQYQT------KNKCTCN----NGD-------------V
1608          QG-YKYFLPK-C-SKDTW--SQ----------DKCRCA----NTG-------------V
0073          RG-NAYFRAT-C--GDEK--KATLA------DVQCRCTKP--NADQ------------V
0060          TG-NKYFRPT-C--GDSG--SPSMA------RDKCRCGDDKKPNDQ------------V
0101          -G-DKYFRAT-C--GGSE--SPSMA------RDKCRC-D----GDQ------------V
0066          GG-NRYFRQT-C--NGGK--S-STP-------NKCRC-DG--ANTD------------P
0191          DG-DTYFRVT-C--NDNG--TLSHA------NHKCRCPNG---NDQ------------V
1617          DG-DTYFRVT-C--SDGR--GGAQA------NNKCRCPNG---KDQ------------V
1621          QN-AKYFRES-F--SDKG--G---T------YDKCRCASG---N--------------V
0058          ES-DKYFRKT-CSGG-K----T-WT------NDKCRCAG----NVR------------V
0130          ND-SQYFRAT-CGSG-RD--AT-RT------RDKCRCAG----NIR------------V
0229          G--SQYFRHT-CGTG-EK--GS-AT------KGKCRCI-----NYD------------V
0075          A--FNYFRHT-CNGG-E------RT------KGYCRCND----DKKPGSNA------DQV
0132          -G-NTYFRAT-CGSDEKN--ST-RT------PNQCRCDG----AKG--AKA------DQV
0181          QG-AQYFHAT-CGSG-NN--AT-RT------PNKCRCGS----YK-------------V
0187          -G-FNYFRHT-CGAG-KD--RT-ET------NHDCQCIN----GD-------------V
1606          NN-AQYFRQT-CGGSGKT--GTL-T------KDKCRCGD----GKKTGTNA------DQV
1622          G--SHYFGHT-CS-KGQG--G---T------QGNCRCAA----G-------------DV
1611          YD-SQYFRAT-CGGDNEN--TTIRT------PSQCRCKDE--NGKKTGTNA------DQV
1887          NN-AQYFRQT-CGGSGKT--GTL-T------KDKCRCGD----GKKTGTNA------DQV
0051          DYAK-YFRGT-CG-SG---KTGTLT------PNQCRCNDGKKPGKNET---------DQV
1613          DYAQ-YFRGT-CG-GDDE-KTGTQA------KNKCRCKD--KNGRPD----------DQV
0126          ANYK-YFRVT-CN-DN---GIFSQA------NDKCRCKK--NDGKNET---------DQV
0004          GGNR-YFRQT-CG-SG---NNPTLA------RDKCRCKN--KKVTNDT---------DQV
0010          SGNN-YFRQT-C--NG-----QHRT------QDNCRCT------THD-----------V
0067          EADK-YFRNA-CS-ND-----TSDT------NEKCRCDG--RNGTNAGKAG---GNVTIV
0109          GGYS-YFRPT-CG-GDN--ESPSVA------NHYCRCND--DNPDDDK--------PNID
0153          RDAS-YFHAT-CN-DTG--QGPSQA------TKQCRCGD--G--D----------VNIV
0078          KDAS-YFRPT-CS-DRK--GSCSQA------KDNCRCD----GSNTDQV-----------
1616          AGAS-YFRAT-CG-GDN--ESPSMA------RDKCRCDDKTSGGKAGEVN--------IV
0017          ASAH-YFRAT-C--DTGQ-GP-SVA------NHYCRCDG--RNGTN----------AHQV
0068          AGAH-YFRAT-CS-DRDRNGTFSQA------NKYCRCDK--EKGAN----------ADQV
0021          ASAS-YFRTT-CS-DT-K-GS-SVA------NNKCRC--------K----------DDQV
0102          SNAS-YFRPT-CS-DR-Q-GE-AQA------KNKCRC--------N----------DNQV
0098          RDAS-YFRPT-C--GSDEKHS-TLA------SNKCRCDK--DKGAK----------ADQV
0228          RDAS-YFRAT-CI-DRNERGQ-SQA------TKQCRCDK--DKGAKD---------GDQV
0064          DNSS-YFRAT-CN-DTG--QGPSQT------HNKCRCDK--DKGANAGKPKAGDGDVTIV
1610          DNSS-YFRPT-C--DTG--KGPSVA------KDHCRCDG--RNGTNADQ----------V
0039          SNAS-YFHAT-CG-DK---KGPSMA------RDKCRCNG--DQ---------------V
0085          DNSNRYFRAT-CDSADG--KGSSVA------RNKCRCKD--EEGKNETN---------EV
0016          -SGT-YFHAT-CSDSDG--NSKSQA------KDKCRCDD-EPSGGKTGKGS---GDVNIV
0174          -HGT-YFHAT-CSDLNG--DC-SQA------HEKCRCP--KTSGGKAIKAG---GDVTIV
0024          -YGT-YFRAT-CSDRNG--SF-SQA------KDKCRCE--KKGGGNVN----------IV
```

```
0168    -HGT-YFRAT-CSERNG--GC-SQA------NDKCRCP--KTSDGKAND---------QV
0046    EGAS-YFRTT-C--SDE--RGGAQA------NDKCRCP--KTSGGKAG-------DVNIV
0083    AGAH-YFRQT-CG-SNA--KTGTQA------SHKCRCP--KTSG-TSG-------DVNID
0061    DYAQ-YFRGT-C--GNT--KIPSKT------PNKCRCP--KTSGGKPGA------NANQV
0043    KNSA-YFRPT-CNG-------GERT------KGYCRCGDGDKPKA--GDGD-----VNIV
0177    ANTH-YFRQT-CNG-------GERT------KGYCRCND-DKPKAVKANDN-----VSIV
1609    ANAS-YFRPT-CGT-------GTGT------QGRCRCDDGKKPGK--ANDN-----VNVV
0202    KNSS-YFRQT-ACA-------GTRT------NDKCRCEG-ANAGK--ANDN-----VNIV
0056    AGAS-YFRPT-CSMNGS----GAQA------RNQCRC-DGA---------------NIV
0089    KNSS-YFRQT-CSDERG----GAQA------NDKCRCPNGN---------------NQV
0059    ASAS-YFRTT-CNDDGT----SSRD------IHKCRCGDGKSNAG-KAGDD-----VNIV
0081    AGGT-YFRTT-ACA-GT----SQTD-------GQCRCAT-----------------NYV
0013    AWGNTYFHAT-CNRGE-R----TKG--------YCRCND--------DQ----------V
0193    AGGNKYFRPT-CGSNE-N--TATQAS------HKCRCND--------DQ----------V
0203    LASASYFRQT-CGGDE-N--TATQAK------NKCRCND--------DQ----------V
0063    --GNAYFRTT-CDSGDNR--GGAQAR------NKCTCNN-------GD-----------V
0198    --SNRYFRQT-CGSGE-K--GSATTG-------KCRCSDKPKSGKPGDVSI--------V
0048    --ESKYFRLT-C-SG-----GNSSAH------NHCTCVD-------GD-----------P
1676    LRGASYFRPT-CNSADGK--GPSQTH------NKCTCNN-------GD-----------V
0015    -GS-QYFRKT-CS--DER--GGAQA------NNYCRCGNDQPNADVPNTD---------P
0149    -GN-AYFRTT-CNSADGR--GGAQA------NNYCRCNGDKPDDDKANTD---------P
0127    -GN-AYFRAT-CS--MNG--SGAQA------NHYCRCNGDQPGQDKTNTD---------P
0148    -NNSSYFHAT-CS--MNG--SGAQA------NKYCRCNGDKPDDDKPNID---------P
0042    -NNSSYFHAT-CS--DGK--SQSQA------NHYCRCNNDQPNADKPNTD---------P
0023    -KNSAYFRVT-CS--DKQ--GESIA------NHKCRCP--MTSDGKPNDQ---------V
0208    -GASQYFRAT-CS-DTGQ--GPSMT------PSQCRCD------DKANTD---------P
0020    -SNASYFRTT-CN-------GGKRT------EGYCRCNGDKPDNDMVNTD---------P
0091    -EDAYYFRQT-C--------GGEKT------ASTNKCR-------CVNTD---------P
0170    -SDK-YFRNT-CN-------DSEHSGTFSQPNKYCRCNGDKPGEDKANVD---------P
0131    -FN--YFRNT-C-----N--GGEGT------KGYCRCNGDKPDDDKANID---------P
0169    -GNA-YFRKT-C-----N--GGEQT------KGYCRCNGDQPGQDRPNTD---------P
1764    -SDK-YFRNT-CS----N--DKAGT------SGKCRCNDNQ------------------V
0215    -DAS-YFRTT-CSD--RQ--GGAQA------RNKCTCNNGD------------------V
0226    -DGS-YFRAT-CGD--SE--SPSQA------RNQCRCQKKDGASV--------------V
0221    -GSQ-YFRAT-CN-------GGERT------KGDCQCAAGD------------------V
0019    -G-SHYFRPT-CST------E-TWSL----D--KCHCANTD------------------V
0223    -DNAQYFRGT-CNY------EGTWSQ----ANHKCRCKKNDG-----KNE------TDQV
0227    -G-GKYFRNT-CAS------LTTWTE----TQGNCQCIGQA------------------V
0040    -GSYKYSQPT-C--------SEEP-L----SQDKCSCPNAG------------------V
0201    -GGGKYFRNT-CGS------AQNPTQ----TEDNCQCIGQA------------------V
0071    -DKAQYFRST-C--------SSKG-G----TYKKCRCINGD------------------V
0052    -GDGKYFRNT-CGE------GTSP------TQNNCRCINFN------------------V
0072    -HGSDYFRHT-CSA------GKYRTA----TNHDCRCINFS------------------V
0079    -SGNNYFRHT-CSD------ENHPTA----TQGNCRCIGAT------------------V
0031    -DNDKYFRNT-CNG------GEQ-------TKDYCRCDDKSKGGKPGKNE------TDQV
0179    -DNAEYFRQT-CGD------STSPSV----AKDHCRCKDEN-GKKTGKNE------SDQV
0134    --VDKYFRKT-CGDD-----SGTLSH----ASNKCRCP-------NGKN-------SDQV
0147    -QNDKYFRQT-CG-------SGEWT------DNNCRC--------DGKN-------AHQV
0133    --GYSYFRPT-CS-D-----GNSKSQ----AKDKCRCEG----------------ANVV
0137    --SYQYSQPT-CS-K------DTLSQ----PK--CSCPN----------------AG-V
0057    --SGTYFRPT-CGGD-----NESPSM----AKNNCRCEG----------------ANVV
0032    ---GTYFRAT-CSDSHR---SETFSQ----AKDNCRC--------DG---------DQV
0107    ---GTYFRAT-CGDEEK---KSTL------AKNNCRCKKE-----DGKSE------TDQA
0008    -G--TYFRAT-CSDSHRSETFSQAKDN-------CRC-DGDQV-----------------
0196    -DDDKYFREK-NSNGN---TCTVNK---------CKCVDGD-P-----------------
0129    -SDDKYFREK-NSEGN---DCFVHK---------CKCANTD-P-----------------
0053    -ADDKYFRKT-ACAGRS----PTDGR--------CRCAANIDP-----------------
0080    -QNDKYFRNT--CAGEH----PTNKQ--------CRCVNLGDV-----------------
0099    -ESDKYFRQT-ACGTGT----PTHKQ--------CRCDDKANVD---------------P
0152    -DDDKYFRTT-AC-AGTR---PTDGQ--------CRCKDDQ-V-----------------
0005    -SDAQYFRNT-CSSI------KGGHY------KNCHCIGGD------------------V
0026    -THSDYFRNT-CSND------KASTY------NKCHCIDGT------------------V
0151    -NDAKYFRKTVCAGG------TTPTH------EKCTCASGD------------------V
0180    -NDAQYFRFT-CLNG------TTRTP------RKCECITHD------------------V
0054    -KDAQYFRNA-CSNG------TTVTD------ARCHCIDQT------------------V
1886    -DGAFYSARG-CCGG------KGSTG------AYCKCANGD------------------V
1618    -DGAFYSARG-CCGG------KGSTG------AYCKCANGD------------------V
1612    -DYAKYFRNA-CTND------TTETD------KKCRCATNY------------------V
0011    -GGSKYFRHT-CGTG------T-PTD------DKCRCAIND------------------V
0037    -VGAQYFGNG-CGDG------KQPTD------TKCRCVSGD------------------P
0049    -TGNKYFRRT-CGTGPD----ASPTQ------GKCRCKTNV------------------V
```

```
0027            -GGDKYFRNT-CAGG------KTPTQ------GKCRCAAGT-----------------V
0034            -NEAKYNVIA-SDGT-----TTKSN------YKKCRNIAD------------------V
0069            -KDAKYKVIK-PDGS-----TKDST------WEKCRDVSD------------------V
0055            -DS-HYTKML-ADGS-----IKQSD------MEKCRNITG------------------V
0104            -NSAHYTKSG-ADGS-----ITEST------MGQCRDVAD------------------V
0030            -RDAHYFLKSSPD--------FKSF------SDL-KCGHYE----------------GAP
0160            -RDAHYFLKSSPD--------FKSF------SDL-KCGHYE----------------GAP
0022            -RDAHYFLKSSPD--------FKSF------SDL-KCGHYE----------------GAP
0171            -RDAHYFLKSSPD--------FKSF------SDL-KCGHYE----------------GAP
0041            -TGADYFKKKSVD--------TVDF------TNDGKCGHTE----------------GTV
1607            -YYADYFKKKSVD--------TVDF------TNDGKCGHNE----------------GAP
0216            -RKADYFRNISKR--------IREF------TDIGKCGHNK----------------GSV
0082            -YDANYVRRKSDR--------TMDF------TSQGQCGRNEN--------------NGYP
0076            -NKSGYFMQ-SKD--------NTLL------SNY-KCGHY----------------EDAP
0214            -PKSAYFLQ-SED--------NKQL------FLYPKCGHNN---------------KNDL
0047            -DKANYFIYKSGN--------TYNF------TTEGYCGRNE----------------RNV
0141            -ENIHYFRKGSDG--------SNLF------SNNGPCGRNE----------------RNV
0197            -ENADYFRKGSDG--------SDVF------SNSGKCGRKE----------------APV
0065            -DSEDYFIQSEGA--------AKSF------SNS-KCGHG----------------EHEV
1614            -GSEEYFIQSENN--------TQLF------SNP-RCGHNK---------------DGDP
0036            -EGDRYSKYLAG---------RITT------VSNEKCGHN----------------DDKV
0084            -ARDEYFKKPSS---------HEYS------FSNGQCGHR----------------DENV
0143            -KDDGYFIKSGY---------RQFK------FTDGHCGRS----------------ETKV
0029            -EEDTYS-KIAD---------DGST------LWNDKCDHN----------------EGIV
0038            -RDAQYFIKSSV---------RDQT------FSNDYCGHD----------------ENKV
0218            -DSEDYFIQSES---------NKKL------FSNSKCGHD----------------ENKV
0050            -AADEYFTKSSN---------LEFE------FTGGYCGRN----------------GEKV
0224            -KEDHYFKPPKN---------RIRE------FTGGYCGRD----------------ETDV
0070            -DNDTYSKYLGVV--------RTT-------LSYPRCGHHV---------------DQDV
0190            -VDDISSRNMGNG--------ITS-------FDF-KCGHN----------------DDNV
0194            -ENDIFSKNIRNS--------RTTL------FDY-KCAHHV---------------NKDV
0173            -HSDEYFRKSTDG--------VTLY------FDG-RCGREL---------------SS-V
0167            -EKAKYFKPPKN---------STRE------FTGGKCGRN----------------EEKV
1615            -EKAHYFKPSEN---------NTQY------FSNKYCGRD----------------EKKV


0003            L-TNFDYVPQYLRW-----
0162            L-TNFDYVPQYLRW-----
0163            L-TNFDYVPQYLRW-----
0007            P-TYFDYVPQYLRWFEEW-
0139            P-TYFDYVPQYLRW-----
0044            P-TYFDYVPQYLRWFDEW-
0002            P-TYFDYVPQYLRW-----
0146            P-TYFDYVPQYLRF-----
0155            P-TYFDYVPQYLRW-----
0110            P-TYFDYVPQYLRW-----
1619            P-TYLDYVPQYLRWFEEWA
0077            P-TYFDYVPQYLRW-----
0200            P-TYLDYVPQFLRWFEEW-
0006            P-TYFDYVPQYLRW-----
0156            P-TYFDYVPQYLRW-----
0028            P-TYFDYVPQYLRW-----
0178            P-TYFDYVPQYLRW-----
0033            P-TYFDYVPQYLRW-----
0100            P-TYFDYVPQYLRW-----
0106            P-TYFDYVPQYLRW-----
0204            P-TYFDYVPQYLRW-----
0138            P-TYFDYVPQYLRW-----
0001            P-TYFDYVPQFLRWFEEW-
1605            P-TYFDYVPQFLRWFEEWA
0012            P-TYFDYVPQYLRWFEEW-
0074            P-TYFDYVPQYLRWF-EW-
1620            P-TYFDYVPQYLRWFEEWA
0014            P-TYFDYVPQYLRWFEEW-
1608            P-TNFDYVPQFLRWFEEWA
0073            P-TSFDYVPQFLRWFEEW-
0060            P-TYFDYVPQFLRWFDEW-
0101            P-TYFDYVPQFLRWFDEW-
0066            P-TYFDYVPQYLRWFDEW-
0191            P-TYFDYVPQFLRWFEEW-
1617            P-TYFDYVPQYLRWFEEWA
```

1621	L-TNFDYVPQYLRWFEEWA
0058	P-TYFDYVPQYLRWFEEW-
0130	P-TYFDYVPQYLRW-----
0229	P-TYFDYVPQYLRWFEEW-
0075	P-TYFDYVPQFLRWFDEW-
0132	P-TYFDYVPQYLRW-----
0181	P-TYFDYVPQYLRW-----
0187	P-TYFDYVPQYLRW-----
1606	P-TYFDYVPQYLRWFEEWA
1622	P-TYFDYVPQYLRWFEEWA
1611	P-TYFDYVPQYLRWFEEWA
1887	P-TYFDYVPQYLRWFEEWA
0051	P-TYFDYVPQYLRWFEEW-
1613	P-TYFDYVPQFLRWFEEWA
0126	P-TYFDYVPQYLRW-----
0004	P-TYFDYVPQYLRWFEEW-
0010	P-TYFDYVPQYLRWFEEW-
0067	P-TYFDYVPQYLRWFEEW-
0109	PPTYFDYVPQYLRW-----
0153	P-TYFDYVPQYLRW-----
0078	P-TYFDYVPQFLRWF-EW-
1616	P-TYFDYVPQYLRWFEEWA
0017	S-TYFDYVPQYLRWFEEW-
0068	P-TYFDYVPQYLRWFEEW-
0021	P-TYFDYVPQYLRWFEEW-
0102	P-TYFDYVPQYLRWFEEW-
0098	P-TYFDYVPQYLRWFDEW-
0228	P-TYFDYVPQYLRWFEEW-
0064	P-TYFDYVPQYLRW-----
1610	P-TYFDYVPQFLRWFEEWA
0039	P-TYFDYVPQYLRWFEEW-
0085	P-TYFDYVPQYLPWFDEW-
0016	P-TYFDYVPQYLRWFEEW-
0174	P-TYFDYVPQYLRWFEEW-
0024	P-TYFDYVPQYLRW-----
0168	P-TYFDYVPQYLRWFDEW-
0046	P-TYFDYVPQYLRW-----
0083	PPTYFDYVPQYLRWFEEW-
0061	P-TYFDYVPQYLRW-----
0043	P-TYFDYVPQYLRWFEEW-
0177	P-TFFDYVPQYLRW-----
1609	P-TYFDYVPQYLRWFEEWA
0202	P-TYFDYVPQFLRW-----
0056	P-TYFDYVPQYLRWFEEW-
0089	P-TYFDYVPQYLRWFEEW-
0059	P-TYFDYVPQYLRWFEEW-
0081	P-TYFDYVPQYLRWFEEW-
0013	P-TYFDYVPQYLRWFEEW-
0193	P-TYFDYVPQFLRWFEEW-
0203	P-TYFDYVPQYLRWFEEW-
0063	P-TYFDYVPQYLRW-----
0198	P-TYFDYVPQYLRW-----
0048	P-TYFDYVPQYLRWFEEW-
1676	P-TYFDYVPQYLRWFEEWA
0015	P-TYFDYVPQYLRWFEEW-
0149	P-TYFDYVPQYLRW-----
0127	P-TYFDYVPQYLRW-----
0148	P-TYFDYVPQYLRW-----
0042	P-TYFDYVPQYLRWFEEW-
0023	P-TYFDYVPQYLRW-----
0208	P-TYFDYVPQYLRWFDEW-
0020	P-TYFDYVPQYLRWFEEW-
0091	P-TYFDYVPQYLRWFEEW-
0170	P-TYFDYVPQYLRWFEEW-
0131	P-TYFDYVPQYLRW-----
0169	P-TYFDYVPQYLRWFEEW-
1764	P-TYFDYVPQYLRWFEEWA
0215	P-TYFDYVPQYLRWFEEW-
0226	P-TYFDYVPQFLRWFEEW-
0221	P-TYFDYVPQYLRW-----
0019	P-TYFDYVPQYLRWFEEW-

```
0223            P-TYFDYVPQYLRWFEEW-
0227            P-TYFDYVPQYLRW-----
0040            P-TNFDYVPQYLRWFDEW-
0201            P-TYFDYVPQYVRWFEEW-
0071            L-TNFDYVPQYLRWFEEW-
0052            P-TYFDYVPQYLRWFEEW-
0072            P-TYFDYVPQYLRWFEEW-
0079            P-TYFDYVPQYLRWFEEW-
0031            P-TYFDYVPQYLRW-----
0179            P-TYFDYVPQYLRW-----
0134            P-TYFDYVPQYLRW-----
0147            P-TYFDYVPQYLRW-----
0133            P-TYFDYVPQYLRW-----
0137            P-TNFDYVPQYLRW-----
0057            P-TYFDYVPQYLRWFDEW-
0032            P-TYFDYVPQYLRW-----
0107            P-TYFDYVPQYLRW-----
0008            P-TYFDYVPQYLRW-----
0196            P-TNLDYVPQYLRW-----
0129            P-TKLDYVPQYLRW-----
0053            P-TYFDYVPQFVRWFEEW-
0080            P-TNFDYVPQYLRWFEEW-
0099            P-TYFDYVPQYLRWFEEW-
0152            P-TYFDYVPQYLRW-----
0005            L-TNFDYVPQYLRW-----
0026            A-TNFDYVPQYLRW-----
0151            P-TYFDYVPQYLRW-----
0180            P-TYFDYVPQYLRW-----
0054            P-TNFDYVPQYLRWFDEW-
1886            P-TNFDYVPQYLRWFEEWA
1618            P-TNFDYVPQYLRWFEEWA
1612            P-TYFDYVPQYLRWFEEWA
0011            P-TYFDYVPQYLRWFEEW-
0037            P-TNFDYVPQYLRW-----
0049            P-TYFDYVPQFLRWFEEW-
0027            P-TYFDYVPQYLRW-----
0034            T-TYFDYVPQYLRW-----
0069            S-TNFDYVPQYLRWFEEW-
0055            P-TNFDYVPQFLRWFEEW-
0104            P-TNFDYVPQYLRW-----
0030            P-TYLDYVPQYLRWF----
0160            P-TYLDYVPQYLRW-----
0022            P-TYLDYVPQYLRW-----
0171            P-TYLDYVPQYLRW-----
0041            P-TNLDYVPQFLRWFDEW-
1607            P-TNLDYVPQFLRWFEEWA
0216            P-TYLDYVPQFLRWFDEW-
0082            L-TNLDYVPQFLRWFDEW-
0076            P-TNLDYVP-YLRWFDEW-
0214            P-TNLDYVPQYLRWFDEW-
0047            P-TYLDYVPQYLRW-----
0141            P-TYLDYVPQYLRW-----
0197            P-TYLDYVPQYLRW-----
0065            L-TNLDYVPQYLRW-----
1614            L-TNLDYVPQFLRWFEEWA
0036            P-TNLDYVPQYLRWFEEW-
0084            P-TNLDYVPQFLRWFDEW-
0143            P-TNLDYVPQYLRW-----
0029            P-TNLDYVPQYLRW-----
0038            L-TNLDYVPQFLRWFEEW-
0218            L-TNLDYVPQFLRWFDEW-
0050            P-TNLDYVPQFLRWFEEW-
0224            P-TNLDYVPQFLRWFDEW-
0070            P-TNLDYVPQHLRWFEEW-
0190            P-TYFDYVPQYLRWFDEW-
0194            Q-TYLDYVPQYLRWFDEW-
0173            P-TYLDYVPQFLRWFDEW-
0167            P-TNLDYVPQYLRWFEEW-
1615            P-TNLDYVPQFLRWFEEWA
                  * :**** .: :
```
