# Supplementary material for: Population Genomics of the Immune Evasion (var) Genes of Plasmodium falciparum
Source: PLoS Pathog. 2007 Mar 16;3(3):e34. doi: 10.1371/journal.ppat.0030034 (PMC1828697; doi:10.1371/journal.ppat.0030034)
Supplement: Supplementary file 2 [file ppat.0030034.sd001.doc]

**Text S1**

***Validation of a sampling framework for the var multigene family***

The population genomic analysis of multigene families such as *var,* in natural populations of *P. falciparum* presents a number of sampling problems: (i) multiple infections are common in endemic areas, requiring that single infections must be identified to ensure that the *var* genes analyzed are from the same genome; (ii) degenerate primers are necessary to amplify the *var* multigene family. The primer bias inherent in PCR amplification using degenerate primers results in the under- or over-representation of variants; (iii) artefacts introduced during PCR reactions may be amplified during the cloning step which is necessary to separate the individual *var* gene sequences of the multigene family; (iv) sampling limitations do not allow identification of complete repertoires; and (v) pseudogenes will also be amplified by degenerate primers and may bias population genomic analysis since they will not be subject to the same selective pressures as functional genes. We addressed these sampling issues by developing a framework for the random epidemiological sampling and population genomic analysis of *var* genes. In this section, we describe the sampling framework.

To amplify DBLα sequences within a genome we used degenerate primers based on conserved amino acid blocks within this domain [1] (Figure S1a). To demonstrate the effectiveness of the degenerate primers we firstly performed PCR with both AFBR [2] and AB primers (see Materials and Methods) using either 3D7 or HB3 genomic DNA as the template. Previously AFBR primers amplified 45 distinct DBLα sequences after sequencing 140 clones (ie: 280 reads) using 3D7 genomic DNA as the template [2,3]. Using our framework (Figure S1a), for 3D7 we obtained 159 reads (from 96 clones sequenced on both strands) for AFBR primers and 193 reads (from 144 clones) for AB primers after quality control; for HB3 we obtained 161 reads for AFBR primers and 150 reads for AB primers. This shows that 16.2-17% (AFBR) and 22-33% (AB) of reads respectively, were lost due to non-specific amplification or low quality sequences. Sequence analysis to remove redundancy (Figure 1b; see Materials and Methods) confirmed the amplification of 9 (3D7) or 21 (HB3) distinct sequences with AFBR and 12 (3D7) or 26 (HB3) with AB primers (Figure S2). The lower number of *var* gene sequences for 3D7 with AFBR primers compared to that of another study [2] may be attributed to fewer clones being sequenced and differences in the PCR conditions. The identity of each of the 3D7 *var* genes [33] obtained was confirmed using BLAST on the PlasmoDB website ([www.plasmodb.org](http://www.plasmodb.org/)). For HB3 we identified *var* genes by using BLAST to search HB3 supercontigs at the Broad Institute website (*Plasmodium falciparum* HB3 Sequencing Project, Broad Institute of Harvard and MIT, http://www.broad.mit.edu). We used the DBLαCIDRα sequence to define structural groups [4,5] for each HB3 *var* gene by drawing a neighbor-joining tree with the 3D7 sequences for which the structural groups were previously defined ([5]; data not shown). Each primer pair amplified a different set of DBLα with some overlap for HB3, but not 3D7 (Figure S2). The uneven distribution of reads among *var* genes shows the inherent primer bias. PCR recombination and amplification of a pseudogene for the AFBR primer set with 3D7 was observed (Figure S2a), but not for the AB primer set with 3D7 (Figure S2c). Both primer sets amplified recombinant or degenerate sequences from HB3 cultured in our laboratory (sample) compared to that from the HB3 genome project (genomic; Figure S2b,d). These changes may have occurred during PCR or during *in vitro* culture. Of note more than double the number of types was obtained with HB3 compared to 3D7, demonstrating the variability in amplification efficiency that occurs even among isolates where DNA template concentration can be controlled. Up to 5 of the 6 DBLαCIDRα *var* gene structural groups [5] were observed for AB primers but only 4 for the AFBR primers (Figure S2). The most highly represented groups were different for 3D7 and HB3 showing that the primers are not biased toward any one group. Although group E was not represented in the 3D7 and HB3 samples for either primer set, it was found within the population sample showing that the primers can amplify this structural group (see main text).

We initially amplified *var* genes from each isolate of the global and local collections (see main text) with both sets of primers and combined the data from 96 clones each because we had obtained limited overlap for the two primer sets in the experiments described above. We found the number of DBLα sequences obtained to be highly variable over the range of high quality sequence reads obtained for each isolate (Figure S3). More than 96 clones (192 reads) were sequenced in 12 of the isolates (for both global and Amele populations), but in only 4 cases this resulting in more sequences than did those with 96 clones or less sampled (Figure S3). Thus, sequencing more than 96 clones infrequently resulted in more sequences. Furthermore, different *var* genes were over-represented in each individual PCR (among isolates) showing that primer bias was apparently random among isolates (Figure S4; Text S2). These results indicated it was not feasible to obtain entire *var* repertoires from each isolate, although it may be possible to define the extent of *var* gene diversity in endemic areas by sampling in depth, ie: more isolates.

**SUPPORTING REFERENCES**

1. Smith JD, Subramanian G, Gamain B, Baruch DI, Miller LH (2000) Classification of adhesive domains in the Plasmodium falciparum erythrocyte membrane protein 1 family. Mol Biochem Parasitol 110: 293-310.

2. Taylor HM, Kyes SA, Harris D, Kriek N, Newbold CI (2000) A study of var gene transcription in vitro using universal var gene primers. Mol Biochem Parasitol 105: 13-23.

3. Kyes S, Taylor H, Craig A, Marsh K, Newbold C (1997) Genomic representation of var gene sequences in Plasmodium falciparum field isolates from different geographic regions. Mol Biochem Parasitol 87: 235-238.

4. Kraemer SM, Smith JD (2003) Evidence for the importance of genetic structuring to the structural and functional specialization of the Plasmodium falciparum var gene family. Mol Microbiol 50: 1527-1538.

5. Lavstsen T, Salanti A, Jensen AT, Arnot DE, Theander TG (2003) Sub-grouping of Plasmodium falciparum 3D7 var genes based on sequence analysis of coding and non-coding regions. Malar J 2: 27.
