# Supplementary material for: Population Genomics of the Immune Evasion (var) Genes of Plasmodium falciparum
Source: PLoS Pathog. 2007 Mar 16;3(3):e34. doi: 10.1371/journal.ppat.0030034 (PMC1828697; doi:10.1371/journal.ppat.0030034)
Supplement: Supplementary file 3 [file ppat.0030034.sd002.doc]

**Text S2**

***Primer bias***

It was of concern that primer bias may result in the under- or over-amplification of some types and thus result in the biased frequencies of types in the population. We therefore compared the average number of sequence reads contributing to each DBLα type, to the spectrum of DBLα type frequencies within each population. In both global and local PNG populations, the average number of sequence reads was variable even for the low type frequencies, and there was only one high frequency type in each population that appeared to be represented by a disproportionate number of reads (Figure S4). Therefore the preferential amplification of types was considered random among isolates and did not appear to favor only the high frequency types.
