# Supplementary material for: Population Genomics of the Immune Evasion (var) Genes of Plasmodium falciparum
Source: PLoS Pathog. 2007 Mar 16;3(3):e34. doi: 10.1371/journal.ppat.0030034 (PMC1828697; doi:10.1371/journal.ppat.0030034)
Supplement: Supplementary file 4 [file ppat.0030034.st002.doc]

**Barry *et al*. Table S2. Properties of Amele DBLα types**

| **DBLα Type Number^a^** | **GenBank Accession Numbers** | **Prevalence** | | **Geographic**  **distribution** | | | | **DBLα group^b^** | ***var* group^c^** | **3D7 match^d^** | **cDNA match^e^** |
| --- | --- | --- | --- | --- | --- | --- | --- | --- | --- | --- | --- |
|  |  | **Amele** | **Global** | **PNG** | **AFRICA** | **AMERICAS** | **ASIA** |  |  |  |  |
| 1736 | DQ13547-48 | 0.03 | 0.02 | x |  |  |  | 4 | n/a |  | nd |
| 84 | DQ135820 | 0.03 | 0.00 | x |  |  |  | 3 | A^f^ |  | nd |
| 101 | DQ135821 | 0.03 | 0.00 | x |  |  |  | 4 | n/a |  | nd |
| 104 | DQ135840 | 0.03 | 0.00 | x |  |  |  | 3 | n/a |  | nd |
| 110 | DQ135841 | 0.03 | 0.00 | x |  |  |  | 4 | n/a |  | nd |
| 127 | DQ135842 | 0.03 | 0.00 | x |  |  |  | 4 | n/a |  | nd |
| 130 | DQ135843 | 0.03 | 0.00 | x |  |  |  | 4 | n/a |  | nd |
| 133 | DQ135844 | 0.03 | 0.00 | x |  |  |  | 4 | n/a |  | nd |
| 134 | DQ135845 | 0.03 | 0.00 | x |  |  |  | 4 | n/a |  | nd |
| 137 | DQ135846 | 0.03 | 0.00 | x |  |  |  | 4 | n/a |  | nd |
| 139 | DQ135847 | 0.03 | 0.00 | x |  |  |  | 4 | n/a |  | nd |
| 140 | DQ135848 | 0.03 | 0.00 | x |  |  |  | 6 | n/a |  | nd |
| 146 | DQ135849 | 0.03 | 0.00 | x |  |  |  | 6 | n/a |  | nd |
| 152 | DQ135850 | 0.03 | 0.00 | x |  |  |  | 4 | n/a |  | nd |
| 155 | DQ135851 | 0.03 | 0.00 | x |  |  |  | 4 | n/a |  | nd |
| 156 | DQ135852 | 0.03 | 0.00 | x |  |  |  | 4 | n/a |  | nd |
| 160 | DQ135853 | 0.03 | 0.00 | x |  |  |  | 3 | A^f^ |  | nd |
| 162 | DQ135854 | 0.03 | 0.00 | x |  |  |  | 4 | n/a |  | nd |
| 163 | DQ135855 | 0.03 | 0.00 | x |  |  |  | 4 | n/a |  | nd |
| 168 | DQ135856 | 0.03 | 0.00 | x |  |  |  | 6 | n/a |  | A4462812 |
| 171 | DQ135857 | 0.03 | 0.00 | x |  |  |  | 3 | A^f^ |  | nd |
| 176 | DQ135858 | 0.03 | 0.00 | x |  |  |  | 4 | n/a |  | nd |
| 177 | DQ135859 | 0.03 | 0.00 | x |  |  |  | 4 | n/a |  | nd |
| 178 | DQ135860 | 0.03 | 0.00 | x |  |  |  | 4 | n/a |  | nd |
| 179 | DQ135861 | 0.03 | 0.00 | x |  |  |  | 4 | n/a |  | nd |
| 180 | DQ135862 | 0.03 | 0.00 | x |  |  |  | 5 | n/a |  | nd |
| 181 | DQ135863 | 0.03 | 0.00 | x |  |  |  | 4 | n/a |  | nd |
| 183 | DQ135864 | 0.03 | 0.00 | x |  |  |  | 6 | n/a |  | nd |
| 184 | DQ135865 | 0.03 | 0.00 | x |  |  |  | 6 | n/a |  | nd |
| 185 | DQ135866 | 0.03 | 0.00 | x |  |  |  | 3 | n/a |  | nd |
| 188 | DQ135867 | 0.03 | 0.00 | x |  |  |  | 6 | n/a |  | nd |
| 191 | DQ135868 | 0.03 | 0.00 | x |  |  |  | 4 | n/a |  | nd |
| 192 | DQ135869 | 0.03 | 0.00 | x |  |  |  | 6 | n/a |  | nd |
| 194 | DQ135870 | 0.03 | 0.00 | x |  |  |  | 2 | A^f^ |  | nd |
| 196 | DQ135871 | 0.03 | 0.00 | x |  |  |  | 4 | n/a |  | nd |
| 198 | DQ135872 | 0.03 | 0.00 | x |  |  |  | 4 | n/a |  | nd |
| 201 | DQ135873 | 0.03 | 0.00 | x |  |  |  | 5 | n/a |  | nd |
| 203 | DQ135874 | 0.03 | 0.00 | x |  |  |  | 4 | n/a |  | nd |
| 208 | DQ135875 | 0.03 | 0.00 | x |  |  |  | 4 | n/a |  | nd |
| 218 | DQ135876 | 0.03 | 0.00 | x |  |  |  | 2 | A^f^ |  | nd |
| 226 | DQ135877 | 0.03 | 0.00 | x |  |  |  | 4 | n/a |  | nd |
| 227 | DQ135878 | 0.03 | 0.00 | x |  |  |  | 4 | n/a |  | nd |
| 228 | DQ135879 | 0.03 | 0.00 | x |  |  |  | 4 | n/a |  | nd |
| 1605 | DQ135822 | 0.03 | 0.00 | x |  |  |  | 4 | n/a |  | nd |
| 1606 | DQ135823 | 0.03 | 0.00 | x |  |  |  | 4 | n/a |  | nd |
| 1607 | DQ135824 | 0.03 | 0.00 | x |  |  |  | 1 | A^f^ |  | nd |
| 1608 | DQ135825 | 0.03 | 0.00 | x |  |  |  | 4 | n/a |  | nd |
| 1609 | DQ135826 | 0.03 | 0.00 | x |  |  |  | 6 | n/a |  | nd |
| 1610 | DQ135827 | 0.03 | 0.00 | x |  |  |  | 6 | n/a |  | nd |
| 1611 | DQ135828 | 0.03 | 0.00 | x |  |  |  | 4 | n/a |  | nd |
| 1612 | DQ135829 | 0.03 | 0.00 | x |  |  |  | 4 | n/a |  | nd |
| 1613 | DQ135830 | 0.03 | 0.00 | x |  |  |  | 5 | n/a |  | nd |
| 1614 | DQ135831 | 0.03 | 0.00 | x |  |  |  | 3 | A^f^ |  | nd |
| 1615 | DQ135832 | 0.03 | 0.00 | x |  |  |  | 3 | n/a |  | nd |
| 1616 | DQ135833 | 0.03 | 0.00 | x |  |  |  | 4 | n/a |  | nd |
| 1617 | DQ135834 | 0.03 | 0.00 | x |  |  |  | 4 | n/a |  | nd |
| 1618 | DQ135835 | 0.03 | 0.00 | x |  |  |  | 6 | n/a |  | nd |
| 1619 | DQ135836 | 0.03 | 0.00 | x |  |  |  | 4 | n/a |  | nd |
| 1620 | DQ135837 | 0.03 | 0.00 | x |  |  |  | 4 | n/a |  | nd |
| 1621 | DQ135838 | 0.03 | 0.00 | x |  |  |  | 6 | n/a |  | nd |
| 1622 | DQ135839 | 0.03 | 0.00 | x |  |  |  | 4 | n/a |  | nd |
| 1872 | DQ135182 | 0.03 | 0.00 | x |  |  |  | 4 | n/a |  | nd |
| 1886 | DQ135209 | 0.03 | 0.00 | x |  |  |  | 6 | n/a |  | nd |
| 1727 | DQ135328-29 | 0.03 | 0.02 | x | x |  |  | 3 | A^f^ |  | nd |
| 1728 | DQ135330-31 | 0.03 | 0.02 | x | x |  |  | 4 | n/a |  | nd |
| 1729 | DQ135332-33 | 0.03 | 0.02 | x |  |  |  | 4 | n/a |  | nd |
| 1730 | DQ135334-35 | 0.03 | 0.02 | x |  |  |  | 4 | n/a |  | nd |
| 1731 | DQ135336-37 | 0.03 | 0.02 | x |  |  |  | 4 | n/a |  | nd |
| 1732 | DQ135338-39 | 0.03 | 0.02 | x | x |  |  | 1 | A^f^ |  | nd |
| 1733 | DQ135340-41 | 0.03 | 0.02 | x |  |  |  | 4 | n/a |  | nd |
| 1734 | DQ135342-43 | 0.03 | 0.02 | x |  |  |  | 2 | A^f^ |  | nd |
| 1735 | DQ135344-45 | 0.03 | 0.02 | x | x |  |  | 4 | n/a |  | nd |
| 1739 | DQ135358-59 | 0.03 | 0.02 | x |  |  |  | 4 | n/a |  | nd |
| 1740 | DQ135360-61 | 0.03 | 0.02 | x |  |  |  | 4 | n/a |  | nd |
| 1744 | DQ135370-71 | 0.03 | 0.02 | x | x |  |  | 1 | A^f^ |  | nd |
| 1745 | DQ135372-73 | 0.03 | 0.02 | x | x |  |  | 4 | n/a |  | nd |
| 1747 | DQ135376-77 | 0.03 | 0.02 | x |  |  |  | 5 | n/a |  | nd |
| 1748 | DQ135378-79 | 0.03 | 0.02 | x |  |  |  | 4 | n/a |  | nd |
| 1749 | DQ135380-81 | 0.03 | 0.02 | x | x |  |  | 1 | A^f^ |  | nd |
| 1751 | DQ135386-87 | 0.03 | 0.02 | x |  |  |  | 2 | A^f^ |  | nd |
| 1752 | DQ135388-89 | 0.03 | 0.02 | x |  |  |  | 3 | A^f^ |  | nd |
| 1753 | DQ135390-91 | 0.03 | 0.02 | x |  |  |  | 4 | n/a |  | nd |
| 1754 | DQ135392-93 | 0.03 | 0.02 | x |  |  |  | 4 | n/a |  | nd |
| 1755 | DQ135394-95 | 0.03 | 0.02 | x |  |  |  | 3 | n/a |  | nd |
| 1761 | DQ135399 | 0.03 | 0.02 | x |  |  |  | 4 | B^h^ | PFE0005w/PF13_0001 | nd |
| 1763 | DQ135402 | 0.03 | 0.02 | x |  |  |  | 4 | D^h^ | PFL0935c | nd |
| 1766 | DQ135408 | 0.03 | 0.02 | x |  |  |  | 2 | A^f,h^ | MAL6P1.316 | nd |
| 1774 | DQ135425 | 0.03 | 0.02 | x |  |  |  | 4 | C^h^ | MAL7P1.56 | nd |
| 1786 | DQ134588 | 0.03 | 0.02 | x |  |  |  | 4 | C^h^ | MAL7P1.50 | nd |
| 1788 | DQ135443 | 0.03 | 0.02 | x |  |  |  | 4 | B^h^ | PFC0005w | nd |
| 1796 | DQ135451 | 0.03 | 0.02 | x |  |  | x | 4 | n/a |  | nd |
| 1807 | DQ135458 | 0.03 | 0.02 | x |  |  | x | 1 | A^f^ |  | nd |
| 1887 | DQ135210-11 | 0.03 | 0.02 | x |  |  |  | 4 | n/a |  | nd |
| 1706 | DQ135281-83 | 0.03 | 0.03 | x |  |  |  | 4 | n/a |  | nd |
| 1722 | DQ135316-18 | 0.03 | 0.03 | x | x |  |  | 4 | n/a |  | nd |
| 1738 | DQ135355-57 | 0.03 | 0.03 | x | x |  |  | 5 | n/a |  | nd |
| 1741 | D1135362-64 | 0.03 | 0.03 | x | x |  |  | 3 | n/a |  | nd |
| 1742 | DQ135365-67 | 0.03 | 0.03 | x |  |  | x | 4 | n/a |  | nd |
| 1743 | DQ135368-69 | 0.03 | 0.03 | x |  |  |  | 6 | n/a |  | nd |
| 1750 | DQ135383-85 | 0.03 | 0.03 | x | x |  |  | 5 | n/a |  | nd |
| 1764 | DQ135403-05 | 0.03 | 0.03 | x | x |  |  | 4 | n/a |  | A4462761 |
| 1737 | DQ135352-53 | 0.03 | 0.05 | x |  |  |  | 4 | n/a |  | nd |
| 1746 | DQ135374-75 | 0.03 | 0.05 | x | x |  |  | 4 | X^h^ | PFL0020w | nd |
| 1691 | DQ135249-51 | 0.03 | 0.07 | x | x |  | x | 4 | n/a |  | nd |
| 1692 | DQ135252-53 | 0.03 | 0.07 | x |  |  | x | 4 | n/a |  | nd |
| 1676 | DQ134581-83 | 0.03 | 0.10 | x | x |  | x | 4 | n/a |  | nd |
| 1697 | DQ135262-65,69,70 | 0.03 | 0.12 | x | x | x | x | 4 | n/a |  | nd |
| 1671^i^ | DQ135233-35 | 0.03 | 0.22 | x | x | x | x | 1 | A^f,g^ |  | A4462739-40 |
| 8 | DQ134134-35 | 0.07 | 0.00 | x |  |  |  | 4 | n/a |  | nd |
| 65 | DQ134417-18 | 0.07 | 0.00 | x |  |  |  | 1 | A^f^ |  | nd |
| 74 | DQ134412-13 | 0.07 | 0.00 | x |  |  |  | 4 | n/a |  | nd |
| 1888 | DQ135212-13 | 0.07 | 0.00 | x |  |  |  | 4 | n/a |  | nd |
| 1889 | DQ135214-215 | 0.07 | 0.00 | x |  |  |  | 4 | n/a |  | nd |
| 1 | DQ134041-43 | 0.07 | 0.02 | x |  |  |  | 4 | n/a |  | nd |
| 14 | DQ134176-78 | 0.07 | 0.02 | x | x |  |  | 4 | n/a |  | nd |
| 15 | DQ134179,81 | 0.07 | 0.02 | x | x |  |  | 4 | n/a |  | nd |
| 41 | DQ134334-36 | 0.07 | 0.02 | x | x |  |  | 1 | n/a |  | A4462745 |
| 43 | DQ134299-300 | 0.07 | 0.02 | x | x |  |  | 4 | n/a |  | nd |
| 49 | DQ134361-63 | 0.07 | 0.02 | x | x |  |  | 4 | n/a |  | nd |
| 52 | DQ134370-72 | 0.07 | 0.02 | x |  |  |  | 4 | n/a |  | nd |
| 57 | DQ134394-96 | 0.07 | 0.02 | x |  |  |  | 4 | n/a |  | nd |
| 60 | DQ134404-06 | 0.07 | 0.02 | x | x |  |  | 4 | n/a |  | nd |
| 61 | DQ134407-09 | 0.07 | 0.02 | x |  |  |  | 4 | n/a |  | nd |
| 64 | DQ134414-16 | 0.07 | 0.02 | x |  |  |  | 4 | n/a |  | nd |
| 70 | DQ134439-41 | 0.07 | 0.02 | x | x |  |  | 2 | A^f^ |  | nd |
| 72 | DQ134446-48 | 0.07 | 0.02 | x |  | x |  | 4 | n/a |  | nd |
| 73 | DQ134450-52 | 0.07 | 0.02 | x | x | x |  | 4 | n/a |  | nd |
| 76 | DQ134459-61 | 0.07 | 0.02 | x | x |  |  | 1 | A^f^ |  | nd |
| 77 | DQ134462-64 | 0.07 | 0.02 | x | x |  |  | 4 | n/a |  | nd |
| 78 | DQ134465-66 | 0.07 | 0.02 | x |  |  |  | 6 | n/a |  | A4462814 |
| 46 | DQ134350-53 | 0.07 | 0.03 | x | x |  |  | 4 | n/a |  | nd |
| 63 | DQ134410-13 | 0.07 | 0.03 | x |  | x |  | 4 | n/a |  | nd |
| 1785 | DQ134585-87,DQ135172 | 0.07 | 0.05 | x | x |  | x | 4 | n/a |  | nd |
| 1682 | DQ135239-41 | 0.07 | 0.07 | x |  |  | x | 5 | n/a |  | nd |
| 34 | DQ134298-303 | 0.07 | 0.10 | x | x |  | x | 3 | n/a |  | nd |
| 12 | DQ134164-66 | 0.10 | 0.00 | x |  |  |  | 4 | n/a |  | nd |
| 28 | DQ134270-72 | 0.10 | 0.00 | x |  |  |  | 4 | n/a |  | nd |
| 42 | DQ134337-39 | 0.10 | 0.00 | x |  |  |  | 4 | n/a |  | nd |
| 45 | DQ134347-49 | 0.10 | 0.00 | x |  |  |  | 3 | n/a |  | nd |
| 48 | DQ134358-60 | 0.10 | 0.00 | x |  |  |  | 4 | n/a |  | nd |
| 50 | DQ134364-66 | 0.10 | 0.00 | x |  |  |  | 1 | A^f^ |  | nd |
| 51 | DQ134367-69 | 0.10 | 0.00 | x |  |  |  | 5 | n/a |  | nd |
| 59 | DQ134401-03 | 0.10 | 0.00 | x |  |  |  | 4 | n/a |  | nd |
| 29 | DQ134273-76 | 0.10 | 0.02 | x | x |  |  | 3 | A^f^ |  | nd |
| 30 | DQ134277-80 | 0.10 | 0.02 | x |  |  |  | 3 | A^f^ |  | nd |
| 33 | DQ134294-97 | 0.10 | 0.02 | x |  |  |  | 4 | n/a |  | nd |
| 39 | DQ134323-26 | 0.10 | 0.02 | x | x |  |  | 4 | n/a |  | nd |
| 44 | DQ134343-46 | 0.10 | 0.02 | x |  | x |  | 4 | n/a |  | nd |
| 47 | DQ134354-57 | 0.10 | 0.02 | x | x |  |  | 1 | A^f^ |  | nd |
| 58 | DQ134397-400 | 0.10 | 0.02 | x | x |  |  | 4 | n/a |  | nd |
| 68 | DQ134430-33 | 0.10 | 0.02 | x | x |  |  | 4 | n/a |  | nd |
| 71 | DQ134442-45 | 0.10 | 0.02 | x | x |  |  | 5 | n/a |  | nd |
| 75 | DQ134455-58 | 0.10 | 0.02 | x |  |  |  | 4 | n/a |  | nd |
| 26 | DQ134259-63 | 0.10 | 0.03 | x |  | x |  | 5 | n/a |  | nd |
| 37 | DQ134312-15 | 0.13 | 0.00 | x |  |  |  | 4 | n/a |  | nd |
| 53 | DQ134373-76 | 0.13 | 0.00 | x |  |  |  | 4 | n/a |  | nd |
| 20 | DQ134224-28 | 0.13 | 0.02 | x | x |  |  | 4 | n/a |  | nd |
| 23 | DQ134246-49 | 0.13 | 0.02 | x |  |  |  | 4 | C^h^ | PFL1960w | A4462813 |
| 27 | DQ134265-69 | 0.13 | 0.02 | x |  |  |  | 4 | n/a |  | nd |
| 32 | DQ134289-93 | 0.13 | 0.02 | x |  |  |  | 4 | n/a |  | nd |
| 56 | DQ134389-93 | 0.13 | 0.02 | x | x |  |  | 4 | n/a |  | nd |
| 66 | DQ134419-23 | 0.13 | 0.02 | x |  | x |  | 4 | n/a |  | nd |
| 69 | DQ134434-38 | 0.13 | 0.02 | x | x |  |  | 3 | n/a |  | nd |
| 3 | DQ134067-72 | 0.13 | 0.03 | x |  | x |  | 4 | n/a |  | nd |
| 55 | DQ134383-87 | 0.17 | 0.00 | x |  |  |  | 3 | n/a |  | nd |
| 19 | DQ134218-23 | 0.17 | 0.02 | x | x |  |  | 4 | n/a |  | nd |
| 21 | DQ134229-34 | 0.17 | 0.02 | x |  |  |  | 6 | n/a |  | nd |
| 31 | DQ134283-88 | 0.17 | 0.02 | x |  |  |  | 4 | n/a |  | nd |
| 54 | DQ134377-82 | 0.17 | 0.02 | x |  |  |  | 4 | n/a |  | nd |
| 67 | DQ134424-29 | 0.17 | 0.02 | x | x |  |  | 5 | n/a |  | nd |
| 38 | DQ134316-22 | 0.20 | 0.02 | x |  |  |  | 2 | A^f^ |  | nd |
| 40 | DQ134327-33 | 0.20 | 0.02 | x | x |  |  | 5 | n/a |  | nd |
| 36 | DQ134304-11 | 0.23 | 0.02 | x |  |  | x | 3 | A^f^ |  | nd |
| 13 | DQ134167-75 | 0.27 | 0.03 | x |  | x | x | 4 | n/a |  | nd |
| 22 | DQ134235-45 | 0.27 | 0.05 | x | x | x |  | 1 | A^f,g^ |  | A4462738 |
| 24 | DQ134250-58 | 0.30 | 0.00 | x |  |  |  | 4 | n/a |  | nd |
| 5 | DQ134084-5,93-104 | 0.30 | 0.12 | x | x | x |  | 4 | n/a |  | nd |
| 4 | DQ134073-83 | 0.33 | 0.02 | x |  | x |  | 6 | C^g^ |  | nd |
| 6 | DQ134508-12,105-10 | 0.33 | 0.02 | x | x |  |  | 4 | n/a |  | nd |
| 16 | DQ134182-93 | 0.37 | 0.02 | x | x |  |  | 4 | A^g^ |  | nd |
| 10 | DQ134136-50 | 0.43 | 0.03 | x | x |  |  | 5 | B^g^ |  | nd |
| 11 | DQ134151-63 | 0.43 | 0.03 | x | x |  |  | 4 | C^g^ E^h^ | PFD1005c | nd |
| 2 | DQ134047-66 | 0.57 | 0.05 | x | x | x |  | 4 | B^g^ |  | nd |
| 7 | DQ134513,515,111-33 | 0.70 | 0.07 | x | x | x |  | 4 | C^g^ |  | nd |
| 17 | DQ134194-217 | 0.77 | 0.02 | x |  |  |  | 4 | B^g^ |  | A4462848 |

^a^The 185 Amele DBLα types are arranged in order of increasing prevalence in the Amele population. ^b^DBLα groups defined using the method of Bull *et al.* 2005 [1]; ^c^*var* gene groups as defined by Lavsten et al. 2003 [2]; ^d^Greater than 96% identity to 3D7 DBLα sequences; ^e^Greater than 96% identity to DBLα sequences derived from cDNA from Maiwara isolates [3] ; ^f^Determined by phylogenetic clustering. 185 Amele DBLα types were translated into amino acid sequences and aligned using Clustal W (Gap opening= 5.0; Gap extension = 0.05) with DBLα sequences from 3D7 *var* genes. A neighbor-joining tree was then constructed using the p-distance algorithm and verified by 1000 bootstrap repetitions. Amele DBLα types that clustered with 3D7 Group A DBLα sequences were listed as Group A *var* genes (see Figure S3a); ^g^Determined by semi-specific PCR using group specific upstream non-coding sequence primers (UpsA, UpsB or UpsC) and DBLα type-specific primers (see Kraemer *et al*. 2004 [4]); ^h^Determined by DBLαCIDRα group of 3D7 var gene [2];  ^i^ >96% identity to *var1csa* gene. Abbreviations: nd = not detected; n/a = not applicable (cannot determine using this method). Shading in the prevalence columns indicates those types that were found in more than 10% of isolates; and in the distribution column the shading highlights the region(s) in which each type was found. Note that 3D7 was considered African for the geographic distribution, as that is its most likely origin.

# REFERENCES

1. Bull PC, Berriman M, Kyes S, Quail MA, Hall N, et al. (2005) Plasmodium falciparum Variant Surface Antigen Expression Patterns during Malaria. PLoS Pathog 1: e26.

2. Lavstsen T, Salanti A, Jensen AT, Arnot DE, Theander TG (2003) Sub-grouping of Plasmodium falciparum 3D7 var genes based on sequence analysis of coding and non-coding regions. Malar J 2: 27.

3. Kaestli M, Cortes A, Lagog M, Ott M, Beck HP (2004) Longitudinal assessment of Plasmodium falciparum var gene transcription in naturally infected asymptomatic children in Papua New Guinea. J Infect Dis 189: 1942-1951.

4. Kraemer SM, Gupta L, Smith JD (2003) New tools to identify var sequence tags and clone full-length genes using type-specific primers to Duffy binding-like domains. Mol Biochem Parasitol 129: 91-102.
